# Leader–follower formation control based on non-inertial frames for non–holonomic mobile robots

**DOI:** 10.1371/journal.pone.0297061

**Published:** 2024-01-29

**Authors:** M. Velasco–Villa, A. Rodriguez–Angeles, I. Z. Maruri–López, J. A. Báez-Hernández, R. D. Cruz Morales

**Affiliations:** 1 Center for Research and Advanced Studies, CINVESTAV–IPN. Electrical Engineering Department, Mechatronics Section, Mexico, Mexico; 2 On sabbatical leave at Eindhoven University of Technology TU/e, Mechanical Engineering Department, Dynamics and Control Group, Eindhoven, The Netherlands; 3 National Autonomous University of Mexico, UNAM FES Cuautitlán, Engineering Department, Electrical Engineering Section, Mexico, México; University of Shanghai for Science and Technology, CHINA

## Abstract

A chain formation strategy based on mobile frames for a set of *n* differential drive mobile robots is presented. Considering two consecutive robots in the formation, robots *R*_*i*_ and *R*_*i*+1_. It is intended that robot *R*_*i*+1_ follows the delayed trajectory, *τ* units of time, of the leader robot *R*_*i*_. In this way, the follower robot *R*_*i*+1_ becomes the leader robot for robot *R*_*i*+ 2_ in the formation and so on. With this formation policy, the trailing distance between two consecutive robots varies accordingly to the velocity of the *R*_*i*_ leader robot. Mobile frames are located on the body of the vehicles, in such a way that the position of robot *R*_*i*_ is determined with respect to the frame located on *R*_*i*+1_ robot. The strategy relies on the fact that the general leader robot *R*_1_ describes any trajectory generated by bounded linear *v*_1_(*t*) and angular *ω*_1_(*t*) velocities. For the remaining vehicles in the string, the strategy considers a desired trajectory for the follower robot *R*_*i*+1_ obtained by an estimation of the delayed trajectory of the leader robot *R*_*i*_. This desired estimated trajectory is obtained under the knowledge of the actual and past input velocities of the *R*_*i*_ robot. To formally prove the convergence of the formation strategy, the equations describing the time variation of the relative posture between any pair of consecutive vehicles in the formation are obtained, and a feedback law based on local measurements is proposed to get the convergence of robot *R*_*i*+1_ to the delayed trajectory, *τ* units of time, of the trajectory previously described by robot *R*_*i*_. Lyapunov techniques are considered for this fact. The effectiveness of the chain formation solution is evaluated by means of numerical simulations and real time experiments showing an adequate convergence.

## Introduction

Nowadays, more applications are using autonomous navigation and the research interest in this area increases day by day due to the complexity of the multi-robots resulting systems, there are several mobile robots applications that take advantage of chain leader-follower formation or platooning strategies to improve traffic performance [1], supply chain [2], or because of safety issues [3]. Either, at street vehicles or small mobile robot applications, a platoon is formed by a leading vehicle and a known or unknown group of follower vehicles, these vehicles may not be aware of all the members that make up the squad, or all the information that comes from them, because, usually each robot has information only from its predecessor.

Platooning formation can be addressed by different approaches, like using a dynamic model based only on position and velocity as if the vehicle were a moving particle [4], meanwhile, others used the kinematic model of the vehicle as a car-like or unicycle robot, when a platoon formation is obtained using the kinematic model it is necessary to consider that these vehicles present a non-holonomic constraint [5, 6], that needs to be taken into account to perform correct navigation. Moreover, when a platoon formation is performed, one of the objectives is to follow the same path for all the mobile robots, sometimes a formation geometric pattern is done at the same time; another specification that can be used is the inter-vehicle distance spacing or time-gap separation policy between vehicles. In order to correctly implement a mobile robot formation, information exchange between robots is necessary, such as position, velocity, acceleration, or heading angle, meanwhile, this information can be obtained either by local or onboard sensors, such as, cameras, LiDAR, GPS, among many others.

A widely used formation for vehicle platooning is the leader-follower formation, using a chain of pairs of leader-follower robots formation, where the first robot is the leader of the one behind it, and then this follower becomes the leader of the robot behind it, this continues until the last robot at the chain. This formation can be performed by different methods depending on the considered reference frame, either local or global. When the mobile robot kinematic model is defined by using a local coordinated frame on the robot body [7], using this mobile frame, sensors onboard like a camera or LiDAR [8, 9], are used to obtain the relative distances and/or the bearing angle between a pair of robots [10, 11] to perform the formation, also, an IMU can be used as a sensor on the robots [12]. In [13], by using a combination of LiDAR, SLAM, and conventional onboard cameras, it is performed the teleoperation of a platoon formation of wheeled mobile robots (WMR) and estimate the current position and predict the future pose of the robots, all of these is done by a fixed distance policy between robots. Another method uses the mobile robot’s local frame and a global frame attached to the earth as a global positioning system (GPS) [14], or some indoor fixed frame to perform the formation [15] using fixed distances, or using a time-gap separation policy between robots [16]. This formation is used for different tasks, as in agriculture [17] where leader–follower formation of two tractors is used to improve the efficiency of the farm by using these robots as rotary cultivators and perform the ground plow more efficiently.

Despite the fact that there are many research related to performing a leader-follower formation or a platoon formation, the main problem is tracking the same path that the leader robot performs, it has been shown that using a fixed distance between robots is not the best method to achieve this objective. For that reason, other methods are proposed, such as speed control by using machine learning [18], where by doing a flexible formation, they maintain a safe varying distance between robots and follow the same straight path, but this is not so useful to perform in curved paths; in [19] they use four different frameworks to perform the platoon formation by controlling velocity, distance, geometry formation, longitudinal and lateral velocities, but this is only used in straight lines and merging operations. In [20] a longitudinal and lateral control strategy is proposed, but the steering strategy is once again only used to change lines, and they proposed that can be extended into a broader driving scenario for future work.

To perform the same path that the leader is tracking, a time-variant spacing policy is proposed in [21], where by using roof cameras the platoon formation is performed, this approach can be useful in open sky scenarios where a GPS signal is strong or where roof cameras can be used and will be always available to track the vehicles from all the area. But in some scenarios the GPS signal loss is an issue, roof cameras are not useful or possible to have, and it is not necessary for all the robots on the platoon to know the position of the rest of them, in these scenarios, a global frame method is not useful. So, there is an option to use a local frame method, where onboard sensors are used to locate other robots and obtain the variables to perform the platoon formation.

In this paper, a platoon formation is performed by using a time-gap separation between robots and assuming that there exist onboard sensors to measure the relative distance and orientation of each pair of consecutive robots. With these measurements, a local frame method is used to obtain the delayed trajectory of the leader robot, wherewith a control law is proposed for the follower robot to perform the same trajectory that the leader robot follows, regardless of its complexity, as long as the leader velocities are assumed to be bounded. Instead of considering the distance and angle between vehicles, the considered sensors must be able to obtain the relative distance, and angle of the leader robot and retain this information to be compared with the actual position of the follower robot, in order to track the delayed trajectory performed by the leader robot. This formation is formally proven by the Lyapunov method and numerical simulations and real–time experiments were considered to show the effectiveness of the platoon formation.

An outline of the article is as follows, first, it is introduced the problem formulation associated with a set of *n* mobile robots type (2,0), represented by their kinematic model. Also, the proposal for the time-varying spacing policy is analyzed. Secondly, the design of an input–delayed observer to generate desired trajectories is presented, and the proposed navigation strategy is described in detail. Then, an evaluation of the presented time–varying spacing policy by means of numerical and real–time experiments are shown, allowing to conclude the article with a discussion of the results and future work.

## Chain formation problem formulation

**Fig 1:**
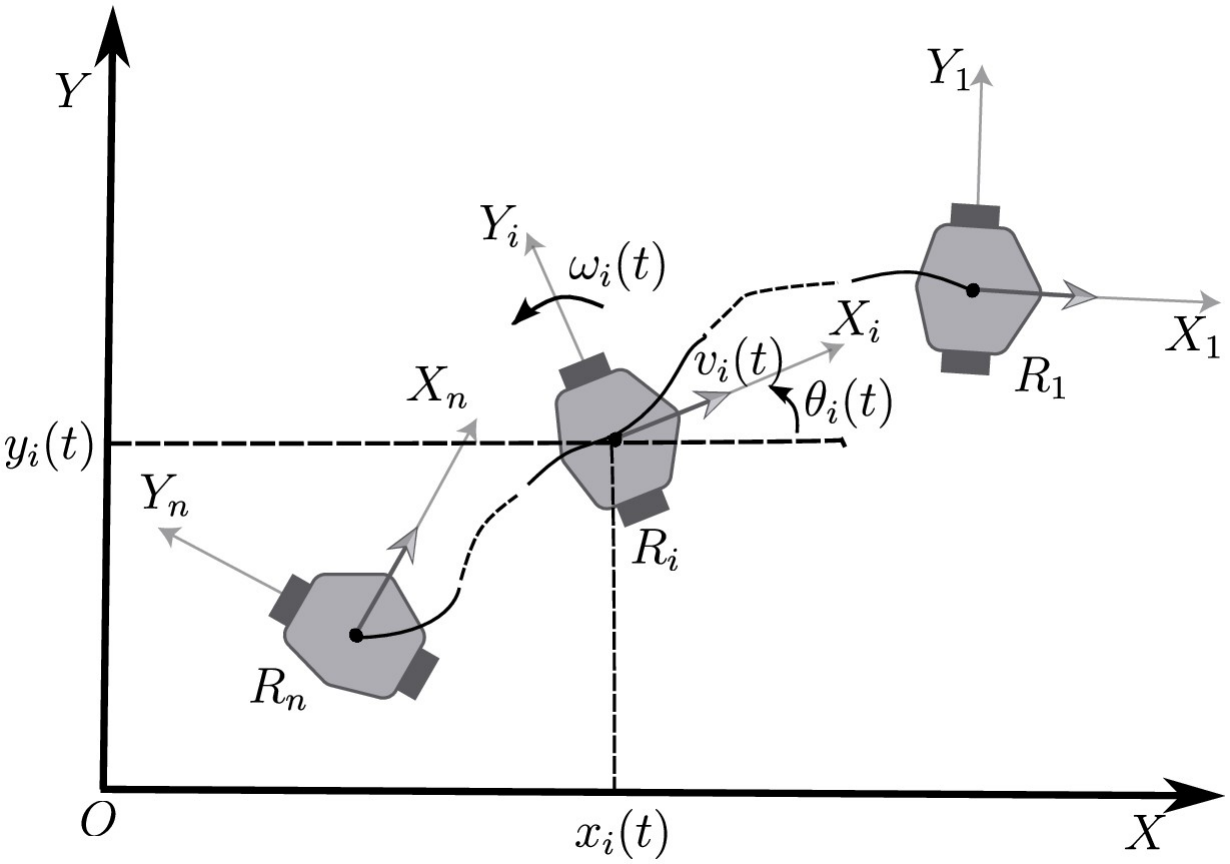
Differential type mobile robot on a fixed frame.

To develop this work, it is considered a set of *n* differential drive mobile robots moving on the *X* − *Y* plane, satisfying non–slipping and non–skidding conditions [22, 23]. The characteristics of these robots are shown in Fig 1 where the *i*-th robot is detailed. The position, at time *t*, of the midpoint of the robot’s wheels axis with respect to the global coordinate frame *X* − *Y* is denoted by the coordinates *x*(*t*) and *y*(*t*), while the orientation of the robot with respect to the *X* axis is denoted by *θ*(*t*). The kinematic model of the *i*-th vehicle [23] can be obtained directly from Fig 1 as,

$$\begin{array}{c} \begin{array}{ccc} {\dot{x}}_{i}\left(t\right) & = & v_{i}\left(t\right)\text{cos}\left(\theta_{i}\left(t\right)\right)\\ {\dot{y}}_{i}\left(t\right) & = & v_{i}\left(t\right)\text{sin}\left(\theta_{i}\left(t\right)\right)\\ {\dot{\theta }}_{i}\left(t\right) & = & \omega_{i}\left(t\right) \end{array} \end{array}$$ (1)

where *u*_*i*_(*t*) = [*v*_*i*_(*t*), *ω*_*i*_(*t*)]^*T*^ are the input signals with *v*_*i*_(*t*) been the linear velocity and *ω*_*i*_(*t*) the angular velocity. The state of the system is given by *ξ*(*t*) = [*x*_*i*_(*t*), *y*_*i*_(*t*), *θ*_*i*_(*t*)]^*T*^. The set of robots is defined by *i* = 1, 2, 3, …, *n* as shown in Fig 1.

Considering that the robots are rigid mechanisms that ideally move on a flat surface, without friction and driven only by the velocities provided by the wheels and that the vertical axes of the wheels are perpendicular to the ground, then, for all time, it is satisfied the non–holonomic constraint [5, 6],

$$\begin{array}{c} {\dot{x}}_{i}\left(t\right)\text{sin}\left(\theta_{i}\left(t\right)\right)-{\dot{y}}_{i}\left(t\right)\text{cos}\left(\theta_{i}\left(t\right)\right)=0. \end{array}$$ (2)

### Formation topology

The objective of this work is to propose a solution to the chain formation problem depicted in Fig 1, without considering a global reference frame for the set of the *n* robots. It is desired to get a solution that allows the extension of the working space of the vehicles without depending on a global positioning system. The platoon formation, depicted in Fig 1 is obtained by numbering the robots from 1 to *n*, starting with the leader robot *R*_1_ through the final robot in the string, *R*_*n*_. It is assumed that the leader robot *R*_1_ describes any trajectory generated by bounded linear *v*_1_(*t*) and angular *ω*_1_(*t*) velocities.

Instead of considering a formation based on the distance and orientation angles between any pair of consecutive robots along the formation [10, 11], in this work, it is desired that the robot *R*_*i*+1_ converges to the path described by the *R*_*i*_ robot delayed a fixed prescribed time. Under these conditions, robot *R*_2_ converges to the delayed trajectory determined by any bounded inputs acting on the leader robot *R*_1_, and robot *R*_3_ converges to the delayed trajectory of robot *R*_2_ and so on. In this way, it is clear that all the robots, let’s say robot *R*_*i*+1_ in the formation, converge to the path described by the leader robot *R*_*i*_ with an adequate time delay. This is, the trajectory performed by robot *R*_*i*_ delayed *τ* units of time will be the desired trajectory that robot *R*_*i*+1_ has to follow.

Notice that solutions depending, for instance, on a GPS strategy suffer from weather conditions, noise present at signals, or, in a different context, indoor working spaces are limited by a local positioning system. Contrary to the above facts, a solution based on local reference frames is more flexible depending only on local measurements that can be easier to obtain. From the above arguments, this work is focused on getting a solution for the described chain formation problem, based on the kinematic models of the robots, where the dynamics of any pair of consecutive robots, let’s say robots *R*_*i*_ and *R*_*i*+1_ in the formation, is described on a local mobile reference frame located over the follower robot *R*_*i*+1_, avoiding the necessity of a global reference frame for all robots.

**Remark 1** *It should be notice that the time τ considered to estimate the delayed trajectory or the leader robot R*_*i*_, *and that will be assumed to be the desired trajectory for the follower robot R*_*i*+1_, *should be determined based on the velocities of the leader robot and the characteristics of the road, and it will be considered as a design parameter. In fact, this time delay can be proposed as in* [24, 25] *where the time gap separation was studied to be optimal in different real traffic scenarios*.

## Chain formation based on mobile frames

As mentioned before, the kinematic model of a mobile robot (1) is developed by considering the measurement of the position and orientation of the vehicle by means of a global positioning system (GPS), or in an indoor working space, by means of an artificial vision system or by odometry measurements in a laboratory. Nevertheless, in a working space where these technological approaches are not possible to implement, the global kinematic models lack the necessary information to be implemented. This latter fact drives this work to consider the development of a kinematic model that does not depend on a global positioning system.

Notice that under the conditions of the formation in Fig 1, for each pair of consecutive robots in the chain formation, robot *R*_*i*_ can be considered as a leader of the follower robot *R*_*i*+1_.

### Kinematic model of the leader–follower formation

**Fig 2:**
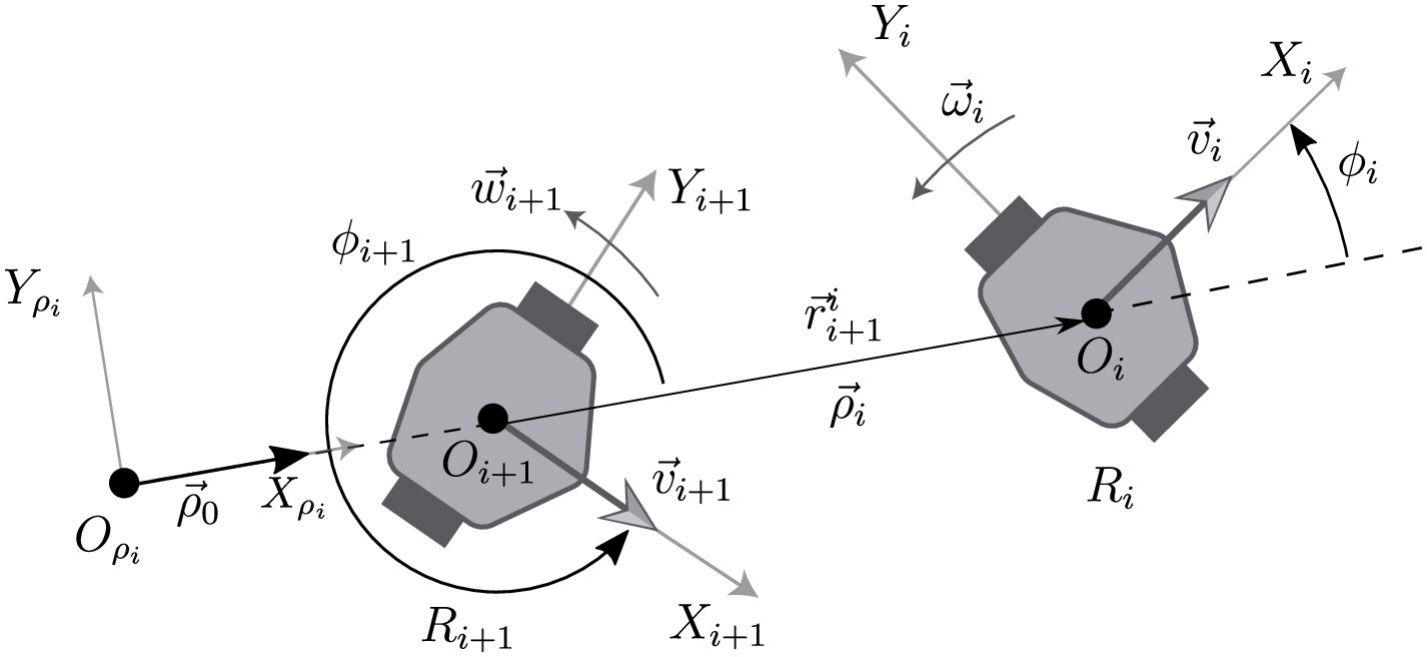
Configuration of a pair of consecutive robots *R*_*i*_ − *R*_*i*+1_ in the chain formation.

To carry out the kinematic model describing the dynamics of a chain formation in mobile frames, consider first, the robot configuration shown in Fig 2. This robot formation can be interpreted as a scenario where considering the notation *S*_*j*_ = *O*_*j*_*X*_*j*_*Y*_*j*_, $S_{\rho_{i}}$ is a general reference frame for the formation, and *S*_*i*_, *S*_*i*+1_ are mobile frames mounted on the center wheel axis of the leader *R*_*i*_ and follower robot *R*_*i*+1_, respectively.

The position of the formation can be determined on the frame $S_{\rho_{i}}$, with this aim, the distance between the robots is given by ${\vec{\rho }}_{i}\left(t\right)$ measured from the origin *O*_*i*+1_ to *O*_*i*_ and the orientation of each robot with respect to ${\vec{\rho }}_{i}\left(t\right)$ is given by *ϕ*_*i*_, *ϕ*_*i*+1_ for the leader *R*_*i*_ and follower *R*_*i*+1_ robot. Therefore, from Fig 2, ${\dot{\vec{\rho }}}_{i}\left(t\right)$ corresponds to the separation time variation between the leader *R*_*i*_ and the follower *R*_*i*+1_ robot on the $S_{\rho_{i}}$ frame, that will be taken as an initial step to get a kinematic model for the configuration that does not depend on a global position system.

From Fig 2, the relative position of the two consecutive robots with respect to frame $S_{\rho_{i}}$ is given as,

$$\begin{array}{c} {\vec{r}}_{i+1}^{i}={\vec{\rho }}_{i}\left(t\right)=\vec{O_{\rho_{i}}O_{i}}\left(t\right)-\vec{O_{\rho_{i}}O_{i+1}}\left(t\right). \end{array}$$ (3)

where the time derivative produces,

$$\begin{array}{c} \frac{d}{dt}{{\vec{r}}_{i+1}^{i}\left(t\right)|}_{S_{\rho_{i}}}=\frac{d}{dt}{\vec{O_{\rho_{i}}O_{i}}\left(t\right)|}_{S_{\rho_{i}}}-\frac{d}{dt}{\vec{O_{\rho_{i}}O_{i+1}}\left(t\right)|}_{S_{\rho_{i}}}. \end{array}$$ (4)

Notice that over its corresponding frames,

$$\begin{array}{c} {{\vec{v}}_{i}|}_{S_{i}}={\left[\begin{array}{ccc} v_{i}\left(t\right) & 0 & 0 \end{array}\right]}^{T}\;\text{and}\;{{\vec{v}}_{i+1}|}_{S_{i+1}}={\left[\begin{array}{ccc} v_{i+1}\left(t\right) & 0 & 0 \end{array}\right]}^{T} \end{array}$$

and therefore, over the frame $S_{\rho_{i}}$ these velocities take the form,

$$\begin{array}{c} {{\vec{v}}_{i}|}_{S_{\rho_{i}}}=R\left(\phi_{i}\right){{\vec{v}}_{i}|}_{S_{i}}\;\text{and}\;{{\vec{v}}_{i+1}|}_{S_{\rho_{i}}}=R\left(\phi_{i+1}\right){{\vec{v}}_{i+1}|}_{S_{i+1}} \end{array}$$

where the rotation matrix *R*(*) in both cases is given as,

$$\begin{array}{c} R\left(✱\right)=[\begin{array}{ccc} \text{cos}(✱) & \text{sin}(✱) & 0\\ \text{sin}(✱) & \text{cos}(✱) & 0\\ 0 & 0 & 1 \end{array}] \end{array}$$ (5)

Under the above conditions, it is possible to get, over the $S_{\rho_{i}}$ frame, the representation,

$$\begin{array}{c} {\frac{d}{dt}{\vec{r}}_{i+1}^{i}\left(t\right)|}_{S_{\rho_{i}}}=[\begin{array}{c} v_{i}\left(t\right)\text{cos}\phi_{i}\left(t\right)-v_{i+1}\left(t\right)\text{cos}\phi_{i+1}\left(t\right)\\ v_{i}\left(t\right)\text{sin}\phi_{i}\left(t\right)-v_{i+1}\left(t\right)\text{sin}\phi_{i+1}\left(t\right)\\ 0 \end{array}]. \end{array}$$ (6)

from where, the relative distance between the robots, on the $S_{\rho_{i}}$ frame has the dynamics,

$$\begin{array}{c} {\dot{\rho }}_{i}\left(t\right)=v_{i}\left(t\right)\text{cos}\phi_{i}\left(t\right)-v_{i+1}\left(t\right)\text{cos}\phi_{i+1}\left(t\right). \end{array}$$ (7)

**Remark 2** *Note that the relation* (7) *does not depend on a global position system, since this velocity variation is independent of the origin* $O_{\rho_{i}}$ on $S_{\rho_{i}}$. *This dynamics is directly related to the linear velocities v*_*i*_(*t*), *v*_*i*+1_(*t*) *of the leader and follower vehicle respectively*.

### Relative model over the *S*_*i*+1_ frame

To simplify the notation over the moving frames, in order to indicate that a vector is referred to the to the *S*_*i*+1_ frame, it will be considered a superscript (*i*+ 1), and a subscript (*i*) to indicate its relation to frame *S*_*i*_. Therefore, a vector between frames *S*_*i*+1_ and *S*_*i*_ measured from frame *S*_*i*+1_ will be denoted as $P_{i}^{i+1}$, with corresponding coordinates given in a similar way as,

$$\begin{array}{c} P_{i}^{i+1}=[\begin{array}{c} x_{i}^{i+1}\left(t\right)\\ y_{i}^{i+1}\left(t\right)\\ z_{i}^{i+1}\left(t\right) \end{array}]. \end{array}$$ (8)

The orientation error between the vehicles is given as,

$$\begin{array}{c} \alpha_{i}^{i+1}\left(t\right)=\phi_{i}\left(t\right)-\phi_{i+1}\left(t\right). \end{array}$$ (9)

The kinematic model referred to the *S*_*i*+1_ frame in Fig 2 will be obtained considering the condition *ρ*_0_ = 0. The posture of the leader robot on *S*_*i*+1_ is obtained in the form,

$$\begin{array}{c} {P_{i}^{i+1}\left(t\right)|}_{S_{i+1}}={R^{T}\left(\phi_{i+1}\left(t\right)\right){\vec{r}}_{i+1}^{i}\left(t\right)|}_{S_{\rho_{i}}}. \end{array}$$ (10)

Also, from Fig 2,

$$\begin{array}{c} \omega_{k}\left(t\right)=\frac{d}{dt}\phi_{k}\left(t\right)\;\text{for}\;k=i,i+1. \end{array}$$

Taking the time derivative of Eq (10),

$$\begin{array}{c} \begin{array}{ccc} {\dot{P}}_{i}^{i+1}\left(t\right) & = & \frac{d}{dt}[R^{T}\left(\phi_{i+1}\left(t\right)\right)]{\vec{r}}_{i+1}^{i}\left(t\right)+R^{T}\left(\phi_{i+1}\left(t\right)\right)\frac{d}{dt}[{\vec{r}}_{i+1}^{i}\left(t\right)]\\ & = & R^{T}\left(\phi_{i+1}\left(t\right)\right)S\left({\dot{\phi }}_{i+1}\left(t\right)\right){\vec{r}}_{i+1}^{i}\left(t\right)+R^{T}\left(\phi_{i+1}\left(t\right)\right)\frac{d}{dt}[{\vec{r}}_{i+1}^{i}\left(t\right)]\\ & = & R^{T}\left(\phi_{i+1}\left(t\right)\right)[{\vec{r}}_{i+1}^{i}\left(t\right)\times {\vec{\omega }}_{i+1}]+R^{T}\left(\phi_{i+1}\left(t\right)\right)\frac{d}{dt}[{\vec{r}}_{i+1}^{i}\left(t\right)]. \end{array} \end{array}$$

Therefore,

$$\begin{array}{c} {\dot{P}}_{i}^{i+1}\left(t\right)=R^{T}\left(\phi_{i+1}\left(t\right)\right)[\frac{d}{dt}[{\vec{r}}_{i+1}^{i}\left(t\right)]-{\vec{\omega }}_{i+1}\times {\vec{r}}_{i+1}^{i}\left(t\right)]. \end{array}$$ (11)

In the above developments, it has been considered the fact that,

$$\begin{array}{c} {\dot{R}}^{T}\left(\phi_{i+1}\left(t\right)\right)=R^{T}\left(\phi_{i+1}\left(t\right)\right)S\left({\dot{\phi }}_{i+1}\left(t\right)\right) \end{array}$$

with $S({\dot{\phi }}_{i+1}\left(t\right))\in SO\left(2\right)$, an skew–symmetric matrix such that,

$$\begin{array}{c} S\left({\dot{\phi }}_{i+1}\left(t\right)\right)=(\begin{array}{ccc} 0 & {\dot{\phi }}_{i+1}\left(t\right) & 0\\ -{\dot{\phi }}_{i+1}\left(t\right) & 0 & 0\\ 0 & 0 & 0 \end{array}). \end{array}$$

After some direct computations, it is possible to write,

$$\begin{array}{c} {\dot{P}}_{i}^{i+1}\left(t\right)=[\begin{array}{c} -v_{i+1}\left(t\right)+v_{i}\left(t\right)\text{cos}(\phi_{i}-\phi_{i+1}\left(t\right))-\omega_{i+1}\left(t\right)\rho_{i}\left(t\right)\text{sin}\phi_{i+1}\left(t\right)\\ v_{i}\left(t\right)\text{sin}(\phi_{i}-\phi_{i+1}\left(t\right))-\omega_{i+1}\left(t\right)\rho_{i}\left(t\right)\text{cos}\phi_{i+1}\left(t\right)\\ 0 \end{array}]. \end{array}$$ (12)

The relative orientation time variation between the leader and follower robot is obtained from Eq (9) as,

$$\begin{array}{c} {\dot{\alpha }}_{i}^{i+1}\left(t\right)=\omega_{i}\left(t\right)-\omega_{i+1}\left(t\right). \end{array}$$ (13)

Notice that, with respect to the frame *S*_*i*+1_, from Eq (12), the relative dynamics between the vehicles depends on the variables $x_{i}^{i+1}\left(t\right)$, $y_{i}^{i+1}\left(t\right)$, *ρ*_*i*_(*t*), *ϕ*_*i*_(*t*) and *ϕ*_*i*+1_(*t*).

It is clear now, that considering Eqs (9) and (13), the relative dynamics on *S*_*i*+1_ is given as,

$$\begin{array}{c} \begin{array}{ccc} {\dot{x}}_{i}^{i+1}\left(t\right) & = & -v_{i+1}\left(t\right)+v_{i}\left(t\right)\text{cos}\alpha_{i}^{i+1}\left(t\right)-\omega_{i+1}\left(t\right)\rho_{i}\left(t\right)\text{sin}\phi_{i+1}\left(t\right)\\ {\dot{y}}_{i}^{i+1}\left(t\right) & = & v_{i}\left(t\right)\text{sin}\alpha_{i}^{i+1}\left(t\right)-\omega_{i+1}\left(t\right)\rho_{i}\left(t\right)\text{cos}\phi_{i+1}\left(t\right)\\ {\dot{\alpha }}_{i}^{i+1}\left(t\right) & = & \omega_{i}\left(t\right)-\omega_{i+1}\left(t\right). \end{array} \end{array}$$ (14)

From Fig 2 it is not difficult to see that,

$$\begin{array}{c} \begin{array}{ccc} x_{i}^{i+1}\left(t\right) & = & \rho_{i}\left(t\right)\text{cos}\left(\phi_{i+1}\left(t\right)\right)\\ y_{i}^{i+1}\left(t\right) & = & -\rho_{i}\left(t\right)\text{sin}\left(\phi_{i+1}\left(t\right)\right) \end{array} \end{array}$$ (15)

that allows to rewrite system (14) to the form,

$$\begin{array}{c} \begin{array}{ccc} {\dot{x}}_{i}^{i+1}\left(t\right) & = & -v_{i+1}\left(t\right)+v_{i}\left(t\right)\text{cos}\alpha_{i}^{i+1}\left(t\right)+\omega_{i+1}\left(t\right)y_{i}^{i+1}\left(t\right)\\ {\dot{y}}_{i}^{i+1}\left(t\right) & = & v_{i}\left(t\right)\text{sin}\alpha_{i}^{i+1}\left(t\right)-\omega_{i+1}\left(t\right)x_{i}^{i+1}\left(t\right)\\ {\dot{\alpha }}_{i}^{i+1}\left(t\right) & = & \omega_{i}\left(t\right)-\omega_{i+1}\left(t\right). \end{array} \end{array}$$ (16)

The kinematic representation of the relative state of the *R*_*i*_ − *R*_*i*+1_ formation (16) can also be obtained by means of alternative procedures, as can be seen in [7, 26].

**Remark 3** *Notice that considering that the follower robot R*_*i*+1_ *is fixed in a point of the working space and that the dynamics of the leader robot R*_*i*_ *is measured on the S*_*i*+1_ *frame, then, under these condition, v*_*i*+1_(*t*) = 0 *and ω*_*i*+1_(*t*) = 0, *for all t*, *and the model* (16) *is rewritten as*,

$$\begin{array}{c} \begin{array}{ccc} {\dot{x}}_{i}^{i+1}\left(t\right) & = & v_{i}\left(t\right)\text{cos}\alpha_{i}^{i+1}\left(t\right)\\ {\dot{y}}_{i}^{i+1}\left(t\right) & = & v_{i}\left(t\right)\text{sin}\alpha_{i}^{i+1}\left(t\right)\\ {\dot{\alpha }}_{i}^{i+1}\left(t\right) & = & \omega_{i}\left(t\right) \end{array} \end{array}$$ (17)

*recovering the representation of the leader robot in a fixed reference frame as in* [23].

**Remark 4** *From* Eq (16) *it is possible to see that the relative orientation* $\alpha_{i}^{i+1}\left(t\right)$ *satisfies the relation*,

$$\begin{array}{c} \text{tan}\alpha_{i}^{i+1}\left(t\right)=\frac{{\dot{y}}_{i}^{i+1}\left(t\right)+\omega_{i+1}\left(t\right)x_{i}^{i+1}\left(t\right)}{{\dot{x}}_{i}^{i+1}\left(t\right)-\omega_{i+1}\left(t\right)y_{i}^{i+1}\left(t\right)+v_{i+1}\left(t\right)} \end{array}$$ (18)

*that in the case that v*_*i*+1_(*t*) = 0 *and ω*_*i*+1_(*t*) = 0, *recovers the non-holonomic constraint* (2).

The solution for the described chain formation, and also, for the leader–follower formation for a pair of robots is obtained under the following assumptions.

**Assumption 1** *The leader robot R*_*i*_ *describes a trajectory generated by bounded linear v*_*i*_(*t*) *and angular ω*_*i*_(*t*) *velocities. This is*,

$$\begin{array}{c} \underset{t\geq t_{0}}{\text{sup}}\left\{v_{i}\left(t\right)\right\}\leq \bar{v_{i}}\;\text{and}\;\underset{t\geq t_{0}}{\text{sup}}\left\{\omega_{i}\left(t\right)\right\}\leq \bar{\omega_{i}} \end{array}$$

*for all t*, *and for* ${\bar{v}}_{i}$, ${\bar{\omega }}_{i}$ ∈*R*. *It is also assumed that for all time either v*_*i*_(*t*) *or ω*_*i*_(*t*) *are not null*.

**Assumption 2** *The follower robot R*_*i*+1_ *has sensors that allow to measure the distance ρ*_*i*_(*t*) *between consecutive robots and the difference between the orientation angles ϕ*_*i*_(*t*) *and ϕ*_*i*+1_(*t*), *where ρ*_*i*_(*t*) ≥ 0 *and* [*ϕ*_*i*_(*t*), *ϕ*_*i*+1_(*t*)] ∈ [−2*π*, 2*π*] ∀ *t*.

**Assumption 3** *The input signals of the robots v*_*k*_(*t*), *ω*_*k*_(*t*) *for k* = *i*, *i* + 1, *in the formation, are available for measurement and they are stored to be shared between the vehicles*.

**Remark 5** *Assumption 1 is a natural condition for any robot, notice that in the case of the first robot in the formation, a feasible trajectory is obtained under any possible physical restriction. Assumption 2, states the technological requirements needed to carry out the proposed formation control. The velocity signals in Assumption 3 are important due to the propagation nature of the chain formation, first based on these signals of the leader robot R*_1_, *its delayed posture is computed, later on, the control signals calculated for robot R*_*i*_ *would be transmitted, taking into account the respective delay, to the robot R*_*i*+1_, *and so on, till robot R*_*n*_.

## Solution for the chain formation problem on mobile frames

As mentioned before, it is intended that a vehicle *R*_*i*+1_ on the chain formation converges to the delayed path described by the precedent robot *R*_*i*_. This can be done by defining as a desired trajectory for the *R*_*i*+1_ robot the estimated delayed trajectory of the leader robot *R*_*i*_. This strategy can be implemented for any pair of consecutive robots *R*_*i*_ − *R*_*i*+1_ in the formation considering robot *R*_*i*_ as the leader and robot *R*_*i*+1_ as the follower.

### Trajectory tracking problem on a mobile frame

As a particular case of the described chain formation problem, when the time delay *τ* between the robots *R*_*i*_ and *R*_*i*+1_ is null, this is, *τ* = 0, it is possible to tackle the trajectory tracking problem for the case of consecutive robots, i.e., when only a pair of robots is considered in the formation. In this case, the robot *R*_*i*_ can be considered as a virtual robot that generates the desired trajectory that robot *R*_*i*+1_ has to track, [22].

Considering the kinematic model of the relative dynamics between two robots on a mobile frame (16), the trajectory tracking problem can be considered by taking the leader robot *R*_*i*_ as a virtual robot that provides the desired reference, that the follower robot *R*_*i*+1_ has to follow, these conditions imply that the states of (16) have to satisfy,

$$\begin{array}{c} \begin{array}{c} {\text{lim}}_{t\to \infty }\left({\dot{x}}_{i}^{i+1}\left(t\right)\right)=0\\ {\text{lim}}_{t\to \infty }\left({\dot{y}}_{i}^{i+1}\left(t\right)\right)=0\\ {\text{lim}}_{t\to \infty }\left({\dot{\alpha }}_{i}^{i+1}\left(t\right)\right)=0. \end{array} \end{array}$$ (19)

Therefore, the trajectory tracking problem for the follower robot becomes a stabilization problem for the kinematic mobile formation (16), when considering the case of consecutive robots, i.e. *R*_*i*_ and *R*_*i*+1_, this is stated in the following lemma.

**Lemma 1** *Assume that Assumptions 1 and 2 are satisfied and consider the leader–follower formation on relative distances given by* (16), *for the case of consecutive robots, i.e. R*_*i*_ *and R*_*i*+1_, *and the feedback*,

$$\begin{array}{c} \begin{array}{ccc} \omega_{i+1}\left(t\right) & = & k_{(i+1)2}\alpha_{i}^{i+1}\left(t\right)+k_{(i+1)3}v_{i}\left(t\right)y_{i}^{i+1}\left(t\right)\frac{\text{sin}\alpha_{i}^{i+1}\left(t\right)}{\alpha_{i}^{i+1}\left(t\right)}+\omega_{i}\left(t\right)\\ v_{i+1}\left(t\right) & = & v_{i}\left(t\right)\text{cos}\alpha_{i}^{i+1}\left(t\right)+k_{(i+1)1}x_{i}^{i+1}\left(t\right) \end{array} \end{array}$$ (20)

*with k*_(*i*+ 1)1_, *k*_(*i*+1)2_ *and k*_(*i*+1)3_ *no null positive gains. Then, if the virtual leader robot is always moving, this is v*_*i*_(*t*) ≠ 0 *or ω*_*i*_(*t*) ≠ 0, *then the states of the closed–loop system* (16)–(20) *asymptotically converge to the origin. Equivalently, the posture of the follower robot converges to that one of the virtual leader robot which generates the desired trajectory*.

**Proof.** To show the result of the lemma, notice first that the closed–loop (16)–(20) takes the form,

$$\begin{array}{c} \begin{array}{ccc} {\dot{x}}_{i}^{i+1}\left(t\right) & = & -k_{(i+1)1}x_{i}^{i+1}+k_{(i+1)2}\alpha_{i}^{i+1}\left(t\right)y_{i}^{i+1}\left(t\right)+k_{(i+1)3}v_{i}\left(t\right){\left(y_{i}^{i+1}\left(t\right)\right)}^{2}\frac{\text{sin}(\alpha_{i}^{i+1}\left(t\right))}{\alpha_{i}^{i+1}\left(t\right)}\\ & & +\omega_{i}\left(t\right)y_{i}^{i+1}\left(t\right)\\ {\dot{y}}_{i}^{i+1}\left(t\right) & = & v_{i}\left(t\right)\text{sin}\alpha_{i}^{i+1}\left(t\right)-k_{(i+1)2}\alpha_{i}^{i+1}\left(t\right)x_{i}^{i+1}\left(t\right)-k_{(i+1)3}x_{i}^{i+1}\left(t\right)v_{i}\left(t\right)y_{i}^{i+1}\frac{\text{sin}\alpha_{i}^{i+1}\left(t\right)}{\alpha_{i}^{i+1}\left(t\right)}\\ & - & \omega_{i}\left(t\right)x_{i}^{i+1}\left(t\right)\\ {\dot{\alpha }}_{i}^{i+1}\left(t\right) & = & -k_{(i+1)2}\alpha_{i}^{i+1}\left(t\right)-k_{(i+1)3}v_{i}\left(t\right)y_{i}^{i+1}\frac{\text{sin}\alpha_{i}^{i+1}\left(t\right)}{\alpha_{i}^{i+1}\left(t\right)}. \end{array} \end{array}$$ (21)

It is clear now that,

$$\begin{array}{c} {\left[\begin{array}{ccc} x_{i}^{i+1} & y_{i}^{i+1} & \alpha_{i}^{i+1} \end{array}\right]}^{T}={\left[\begin{array}{ccc} 0 & 0 & 0 \end{array}\right]}^{T} \end{array}$$ (22)

is an equilibrium point of the closed–loop system (21).

Consider now, the following candidate Lyapunov function,

$$\begin{array}{c} V\left(t\right)=\frac{k_{(i+1)3}}{2}\left({\left(x_{i}^{i+1}\left(t\right)\right)}^{2}+{\left(y_{i}^{i+1}\left(t\right)\right)}^{2}\right)+\frac{1}{2}{\left(\alpha_{i}^{i+1}\left(t\right)\right)}^{2}. \end{array}$$ (23)

The time derivative of (23) produces, after a simple procedure,

$$\begin{array}{c} \dot{V}\left(t\right)=-k_{(i+1)1}k_{(i+1)3}{\left(x_{i}^{i+1}\left(t\right)\right)}^{2}-k_{(i+1)2}{\left(\alpha_{i}^{i+1}\left(t\right)\right)}^{2} \end{array}$$

that shows the stability of the closed–loop system.

The asymptotic stability of the system (21), follows by considering the Barbalat’s Lemma [27]. In this sense, notice that $\ddot{V}\left(t\right)$ can be computed as,

$$\begin{array}{cc} \ddot{V}\left(t\right) & =-2k_{(i+1)1}k_{(i+1)3}x_{i}^{i+1}{\dot{x}}_{i}^{i+1}-2k_{(i+1)2}\alpha_{i}^{i+1}{\dot{\alpha }}_{i}^{i+1}\\ & =-2k_{(i+1)i}k_{(i+1)3}x_{i}^{i+1}[-k_{(i+1)1}x_{i}^{i+1}+k_{(i+1)2}\alpha_{i}^{i+1}y_{i}^{i+1}+k_{(i+1)3}v_{i}{\left[y_{i}^{i+1}\right]}^{2}\frac{\text{sin}\alpha_{i}^{i+1}}{\alpha_{i}^{i+1}}\\ & +\omega_{i}y_{i}^{i+1}]-2k_{(i+1)2}\alpha_{i}^{i+1}\left[-k_{(i+1)2}\alpha_{i}^{i+1}-k_{(i+1)3}v_{i}y_{i}^{i+1}\frac{\text{sin}\alpha_{i}^{i+1}}{\alpha_{i}^{i+1}}\right] \end{array}$$

Therefore, $\ddot{V}\left(t\right)$ is a function of the variables $x_{i}^{i+1}\left(t\right)$, $y_{i}^{i+1}\left(t\right)$ and $\alpha_{i}^{i+1}\left(t\right)$ that are bounded since $\dot{V}\left(t\right)\leq 0$ and the velocities *v*_*i*_(*t*) and *ω*_*i*_(*t*) are also bounded by Assumption 1. Then, $\dot{V}\left(t\right)$ is a uniformly continuous function, from where, $\dot{V}\left(t\right)\to 0$ as *t* → ∞ and consequently, $x_{i}^{i+1}\left(t\right)\to 0$, $\alpha_{i}^{i+1}\left(t\right)\to 0$ as *t* → ∞.

From the third equation in (21), and from the convergence of $\alpha_{i}^{i+1}\left(t\right)$ to the origin,

$$\begin{array}{c} k_{(i+1)3}v_{i}\left(t\right)y_{i}^{i+1}\left(t\right)=0 \end{array}$$

this is,

$$\begin{array}{c} v_{i}\left(t\right)y_{i}^{i+1}\left(t\right)=0. \end{array}$$ (24)

Also, notice that from the first equation in (21), it is obtained,

$$\begin{array}{c} k_{(i+1)3}v_{i}\left(t\right){\left(y_{i}^{i+1}\left(t\right)\right)}^{2}+\omega_{i}\left(t\right)y_{i}^{i+1}\left(t\right)=0 \end{array}$$

that considering Eq (24) it is produced,

$$\begin{array}{c} \omega_{i}\left(t\right)y_{i}^{i+1}\left(t\right)=0. \end{array}$$ (25)

Therefore, considering again Assumption 1, from Eqs (24) and (25), the result of the lemma is stated.

### Delayed trajectory tracking problem for the chain formation problem

Instead of considering a distance ${\vec{\rho }}_{i}$ and angle *ϕ* = *π* − *ϕ*_*i*_ between a pair of consecutive robots in the platoon, see Fig 2, as is usual in this type of formation, it is desired that the follower robot *R*_*i*+1_ tracks the trajectory described by the leader robot *R*_*i*_.

**Fig 3:**
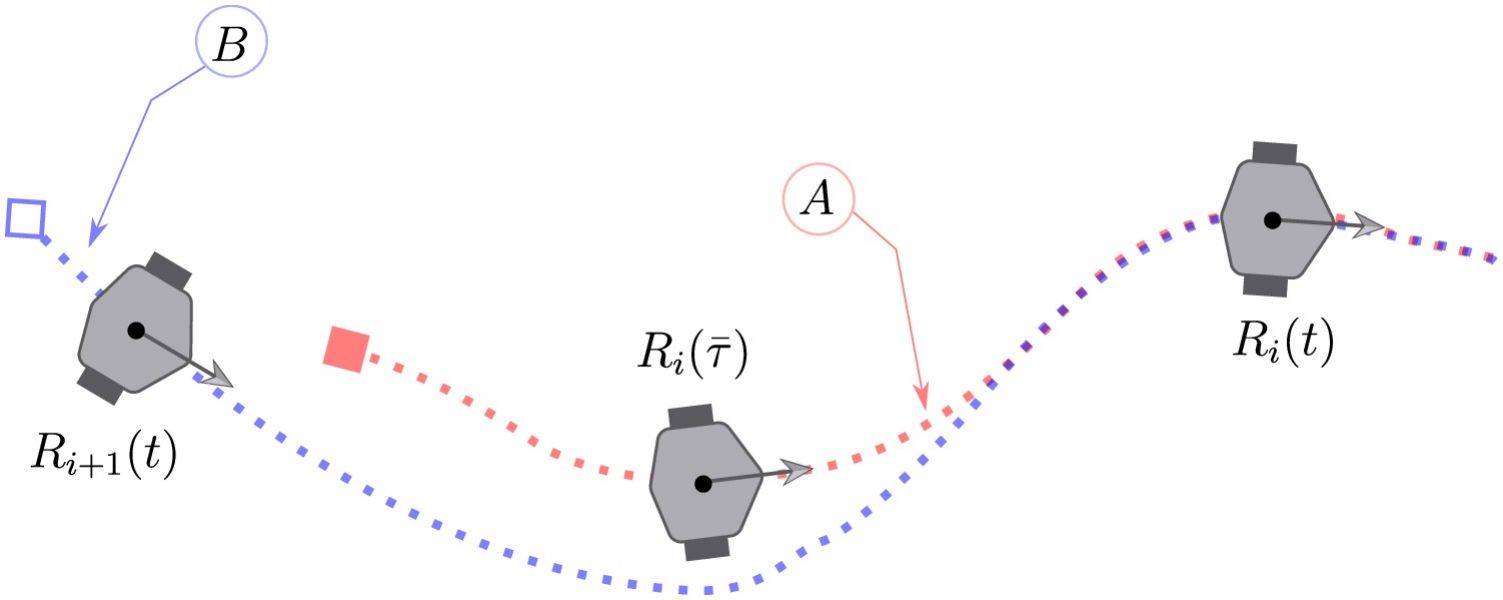
Tracking of the delayed leader robot *R*_*i*_ trajectory.

For a pair of consecutive robots in the chain formation, to design a feedback that allows the robot *R*_*i*+1_ to track the trajectory described by the robot *R*_*i*_ obtained by means of bounded inputs *v*_*i*_(*t*) and *ω*_*i*_(*t*), consider the configuration depicted in Fig 3, where it is shown the trajectory, marked as *A*, of the leader robot *R*_*i*_(*t*) together with its delayed image $R_{i}\left(\bar{\tau }\right)$ for $\bar{\tau }=t-\tau$, and the trajectory *B* of the follower robot *R*_*i*+1_(*t*). It is desired that the follower robot *R*_*i*+1_ converges to the delayed trajectory (dotted red line) of the leader robot $R_{i}\left(\bar{\tau }\right)$ as *t* tends to infinity.

**Fig 4:**
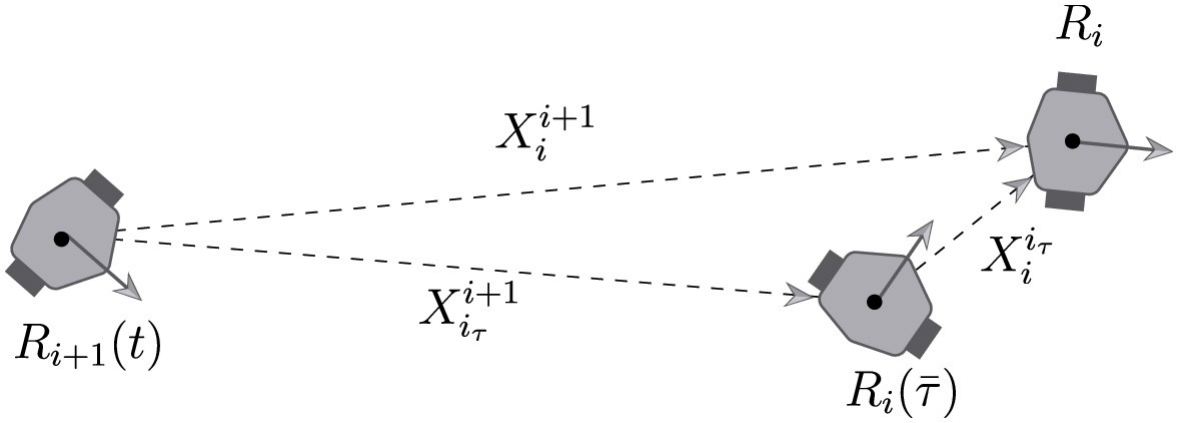
Position configuration of the leader robot *R*_*i*_(*t*), its delayed image $R_{i}\left(\bar{\tau }\right)$ and the follower robot *R*_*i*+1_(*t*).

To propose a solution to the chain formation problem consider now the robot’s configuration shown in Fig 4 where the leader robot *R*_*i*_(*t*), its *τ* units of time delayed image $R_{i}\left(\bar{\tau }\right)$, and the follower robot *R*_*i*+1_(*t*) are described.

As described before, in Fig 4, $X_{i}^{i+1}\left(t\right)$ corresponds to the measurement of the posture of the leader robot *R*_*i*_ with respect to the mobile axes located on the follower robot *R*_*i*+1_, i.e. on the frame *S*_*i*+1_, by means of onboard sensors of the *R*_*i*+1_ robot as described in Assumption 2. $X_{i_{\tau }}^{i+1}\left(t\right)$ corresponds to the posture of the virtual delayed leader robot $R_{i}\left(\bar{\tau }\right)$ measured from the follower robot *R*_*i*+1_ refereed to *S*_*i*+1_, and $X_{i}^{i_{\tau }}\left(t\right)$ corresponds to the position of the leader robot refereed to a frame mounted on the virtual delayed leader robot $R_{i}\left(\bar{\tau }\right)$.

First of all, it is assumed that the vector $X_{i}^{i+1}$ is directly obtained by the sensor mounted on the *R*_*i*+1_ robot. The dynamics of $X_{i}^{i_{\tau }}\left(t\right)$ can be obtained easily by considering Assumption 3 and noticing that the leader–follower configuration between $R_{i}\left(\bar{\tau }\right)$ and *R*_*i*_(*t*) can be obtained directly from Eq (16) taking into account the measurement of the input velocities, *v*_*i*_(*t*) and *ω*_*i*_(*t*) in the form,

$$\begin{array}{c} \begin{array}{ccc} {\dot{x}}_{i}^{i_{\tau }}\left(t\right) & = & -v_{i}\left(\bar{\tau }\right)+v_{i}\left(t\right)\text{cos}\alpha_{i}^{i_{\tau }}\left(t\right)+\omega_{i}\left(\bar{\tau }\right)y_{i}^{i_{\tau }}\left(t\right)\\ {\dot{y}}_{i}^{i_{\tau }}\left(t\right) & = & v_{i}\left(t\right)\text{sin}\alpha_{i}^{i_{\tau }}\left(t\right)-\omega_{i}\left(\bar{\tau }\right)x_{i}^{i_{\tau }}\left(t\right)\\ {\dot{\alpha }}_{i}^{i_{\tau }}\left(t\right) & = & \omega_{i}\left(t\right)-\omega_{i}\left(\bar{\tau }\right). \end{array} \end{array}$$ (26)

The state of the virtual dynamics (26) will be considered as a desired behavior for the state $X_{i_{\tau }}^{i+1}$.

From the robot’s configuration in Fig 4, considering the relative dynamics (26) and the measured signal $X_{i}^{i+1}\left(t\right)$, it is possible to get the state vector $X_{i_{\tau }}^{i+1}\left(t\right)$ in the form,

$$\begin{array}{c} X_{i_{\tau }}^{i+1}\left(t\right)=X_{i}^{i+1}\left(t\right)-R\left(\gamma \left(t\right)\right)X_{i}^{i_{\tau }}\left(t\right) \end{array}$$ (27)

where,

$$\begin{array}{c} \gamma \left(t\right)=\alpha_{i}^{i+1}\left(t\right)-\alpha_{i}^{i_{\tau }}\left(t\right) \end{array}$$ (28)

with the rotation matrix *R*(*) given by (5).

Also notice that the dynamics of $X_{i_{\tau }}^{i+1}\left(t\right)$ is described by means of the equations,

$$\begin{array}{c} \begin{array}{ccc} {\dot{x}}_{i_{\tau }}^{i+1}\left(t\right) & = & -v_{i+1}\left(t\right)+v_{i}\left(\bar{\tau }\right)\text{cos}\alpha_{i_{\tau }}^{i+1}\left(t\right)+\omega_{i+1}\left(t\right)y_{i_{\tau }}^{i+1}\left(t\right)\\ {\dot{y}}_{i_{\tau }}^{i+1}\left(t\right) & = & v_{i}\left(\bar{\tau }\right)\text{sin}\alpha_{i_{\tau }}^{i+1}\left(t\right)-\omega_{i+1}\left(t\right)x_{i_{\tau }}^{i+1}\left(t\right)\\ {\dot{\alpha }}_{i_{\tau }}^{i+1}\left(t\right) & = & \omega_{i}\left(\bar{\tau }\right)-\omega_{i+1}\left(t\right). \end{array} \end{array}$$ (29)

**Remark 6** *The stabilization of* Eq (29) *provides the convergence of the follower robot R*_*i*+1_ *to the delayed trajectory of the leader robot R*_*i*_. *This fact allows that, in the chain formation, the robot R*_2_ *follows the delayed trajectory of robot R*_1_, *that R*_3_ *tracks the delayed trajectory of R*_2_, *and so on, producing that at the rear of the formation, robot R*_*n*_ *follows the delayed trajectory of robot R*_*n*−1_.

The convergence of the *R*_*i*+1_ robot to the delayed trajectory of the *R*_*i*_ robot is stated in the next theorem.

**Theorem 1** *Consider the relative dynamics* (29) and that Assumptions 1, 2 and 3 are satisfied. Under the configuration shown in Figs 3 and 4, the feedback,

$$\begin{array}{c} \begin{array}{ccc} \omega_{i+1}\left(t\right) & = & k_{(i+1)2}\alpha_{i_{\tau }}^{i+1}\left(t\right)+k_{(i+1)3}y_{i_{\tau }}^{i+1}\left(t\right)v_{i}\left(\bar{\tau }\right)\frac{\text{sin}\alpha_{i_{\tau }}^{i+1}\left(t\right)}{\alpha_{i_{\tau }}^{i+1}\left(t\right)}+\omega_{i}\left(\bar{\tau }\right)\\ v_{i+1}\left(t\right) & = & v_{i}\left(\bar{\tau }\right)\text{cos}\alpha_{i_{\tau }}^{i+1}\left(t\right)+k_{(i+1)1}x_{i_{\tau }}^{i+1}\left(t\right) \end{array} \end{array}$$ (30)

*with k*_(*i*+ 1)1_, *k*_(*i*+1)2_ *and k*_(*i*+1)3_ *positive constant gains, makes the R*_*i*+1_ *robot asymptotically tracks the delayed trajectory R*_*i*_(*t* − *τ*) *of the leader robot. This is, feedback* (30) asymptotically stabilizes the dynamics of the system (29).

**Proof.** Notice that the closed–loop system (29) and (30) produces,

$$\begin{array}{lll} {\dot{x}}_{i_{\tau }}^{i+1}(t) & = & \begin{array}{l} -k_{(i+1)1}x_{i_{\tau }}^{i+1}(t)+k_{2}\alpha_{i_{\tau }}^{i+1}(t)y_{i_{\tau }}^{i+1}(t)+k_{(i+1)3}{\left(y_{i_{\tau }}^{i+1}(t)\right)}^{2}(t)v_{i}(\bar{\tau })\frac{\text{sin}\alpha_{i_{\tau }}^{i+1}(t)}{\alpha_{i_{\tau }}^{i+1}(t)}+\\ \omega_{i}(\bar{\tau })y_{i_{\tau }}^{i+1}(t) \end{array}\\ {\dot{y}}_{i_{\tau }}^{i+1}(t) & = & \begin{array}{l} v_{i}(\bar{\tau })\text{sin}\alpha_{i_{\tau }}^{i+1}(t)-k_{(i+1)2}\alpha_{i_{\tau }}^{i+1}(t)x_{i_{\tau }}^{i+1}(t)-k_{(i+1)3}x_{i_{\tau }}^{i+1}(t)y_{i_{\tau }}^{i+1}(t)v_{i}(\bar{\tau })\frac{\text{sin}\alpha_{i_{\tau }}^{i+1}(t)}{\alpha_{i_{\tau }}^{i+1}(t)}\\ -\omega_{i}(\bar{\tau })x_{i_{\tau }}^{i+1}(t) \end{array}\\ {\dot{\alpha }}_{i_{\tau }}^{i+1}(t)\; & = & -k_{(i+1)2}\alpha_{i_{\tau }}^{i+1}(t)-k_{(i+1)3}y_{i_{\tau }}^{i+1}(t)v_{i}(\bar{\tau })\frac{\text{sin}\alpha_{i_{\tau }}^{i+1}(t)}{\alpha_{i_{\tau }}^{i+1}(t)}. \end{array}$$ (31)

Then, it is clear that,

$$\begin{array}{c} \left[\begin{array}{ccc} x_{i_{\tau }}^{i+1} & y_{i_{\tau }}^{i+1} & \alpha_{i_{\tau }}^{i+1} \end{array}]=[\begin{array}{ccc} 0 & 0 & 0 \end{array}\right] \end{array}$$

is an equilibrium point for the system (21).

To complete the proof, it should be noticed that Eq (31) has the same structure as Eq (21) in the proof of Lemma 1. Therefore, it is clear that a candidate Lyapunov function of the form,

$$\begin{array}{c} V_{1}\left(t\right)=\frac{k_{(i+1)3}}{2}\left({\left(x_{i}^{i+1}\left(t\right)\right)}^{2}+{\left(y_{i}^{i+1}\left(t\right)\right)}^{2}\right)+\frac{1}{2}{\left(\alpha_{i}^{i+1}\left(t\right)\right)}^{2}. \end{array}$$ (32)

produces the time derivative of (32),

$$\begin{array}{c} {\dot{V}}_{1}\left(t\right)=-k_{(i+1)1}k_{(i+1)3}{\left(x_{i_{\tau }}^{i+1}\left(t\right)\right)}^{2}-k_{(i+1)2}{\left(\alpha_{i_{\tau }}^{i+1}\left(t\right)\right)}^{2} \end{array}$$

that shows the stability of the closed–loop system (29) and (30).

It is clear now, that asymptotic stability of the system (29) is shown following the lines of Lemma 1.

**Remark 7** *It should be pointed out that the delayed trajectory strategy, in order to follow the leader’s delayed path, has been previously considered in* [16, 21] *where the strategy is developed in a different context by referring the kinematic model of the robots to a global reference frame*.

## Performance evaluation

The evaluation of the proposed strategy will be carried out by considering numerical simulations and real–time experiments for the trajectory tracking problem (16)–(20), and later for the leader delayed chain formation strategy, (29) and (30). For the leader robot, it is considered a lemniscate type trajectory generated by bounded linear *v*_1_(*t*) and rotational *ω*_1_(*t*) velocities. For the tracking performance test only a pair of robots is considered, i.e. *n* = 2, and the time delay is set as zero *τ* = 0; meanwhile for the platooning case, three robots are taken into account.

### Lemniscate type desired trajectory generation

Since the first robot in the formation can perform any trajectory produced by the action of bounded input velocities, to generate a specific desired trajectory for the leader robot in the chain formation, it will be considered a path obtained by input velocity signals defined in the form,

$$\begin{array}{c} \begin{array}{ccc} v_{1}\left(t\right) & = & \sqrt{{\dot{x}}_{d}^{2}\left(t\right)+{\dot{y}}_{d}^{2}\left(t\right)}\\ \omega_{1}\left(t\right) & = & \frac{{\ddot{y}}_{d}\left(t\right){\dot{x}}_{d}\left(t\right)-{\ddot{x}}_{d}\left(t\right){\dot{y}}_{d}\left(t\right)}{{\dot{x}}_{d}^{2}\left(t\right)+{\dot{y}}_{d}^{2}\left(t\right)} \end{array} \end{array}$$ (33)

where *x*_*d*_(*t*) and *y*_*d*_(*t*) correspond to signals obtained by a lemniscate type trajectory on the *X* − *Y* plane given as,

$$\begin{array}{c} \begin{array}{ccc} x_{d}\left(t\right) & = & a\;\text{cos}(pt)\\ y_{d}\left(t\right) & = & b\;\text{sin}(2pt) \end{array} \end{array}$$ (34)

with *a* = 0.8, *b* = 0.6 and $p=\frac{2\pi }{50}$. Notice that a specific path for the virtual or the leader robot is not necessary for the experiments, bounded velocity inputs is the only requirement.

### Numerical trajectory tracking evaluation

For the tracking evaluation only a pair of robots is considered, i.e. *n* = 2, and the time delay is set as zero *τ* = 0, thus robot *R*_2_ has to track the exact time trajectory of robot *R*_1_. The numerical simulation for the trajectory tracking problem is carried out by considering the initial conditions given in Table 1, where they are referred to a global frame and to the moving reference frame *S*_2_, that is considered to develop the strategy of this work. For easy understanding and comparison of results, a global reference frame is used to present the time evolution of the tracking errors along the chain formation, nevertheless, the control actions (20) for trajectory tracking, and (30) for chain formation, make use only of local frame measurements. The gains considered for the feedback (20) were set as *k*_21_ = 2, *k*_22_ = 3 and *k*_23_ = 2.

**Table 1:** Initial conditions for the trajectory tracking evaluation.

	Virtual *R*_1_	Robot *R*_2_
Global Frame	*x*_1_ = 0 *y*_1_ = 0 *θ*_1_ = 0	*x*_2_ = −0.2 *y*_2_ = −0.3 $\theta_{2}=\frac{3\pi }{2}$
Moving Frame *S*_2_, *S*_3_	$x_{1}^{2}=-0.3$ $y_{1}^{2}=0.2$ $\alpha_{1}^{2}=-\frac{3\pi }{2}$	

**Fig 5:**
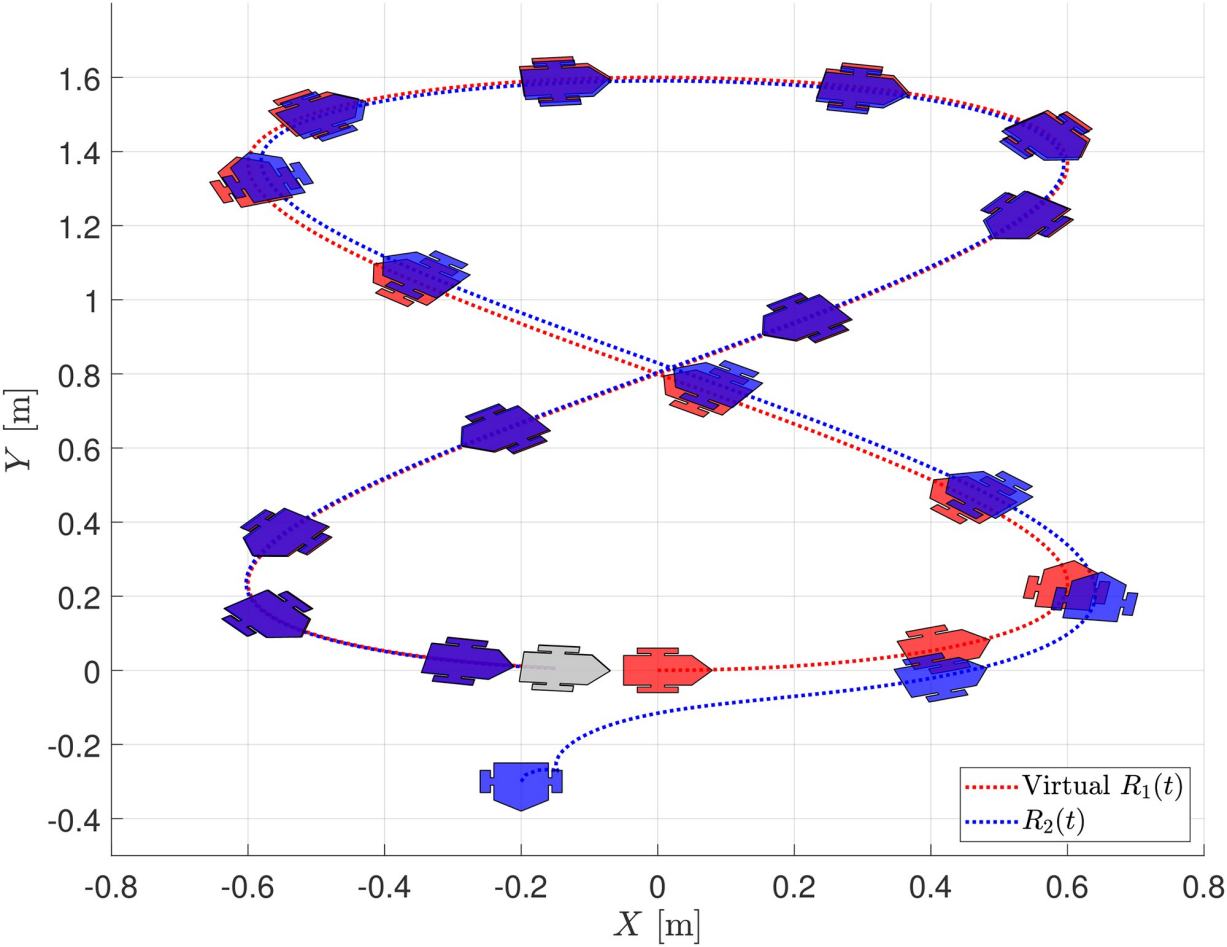
Trajectory tracking evolution on the plane.

Assuming that it is possible to measure the displacement of the robot on the global reference frame, Fig 5 shows the time evolution of the robot on the *X* − *Y* plane. Notice how the follower robot *R*_2_ (blue) converges to the desired trajectory defined by the virtual leader robot *R*_1_ (red). The final position of the two robots is depicted in gray for the final time.

**Fig 6:**
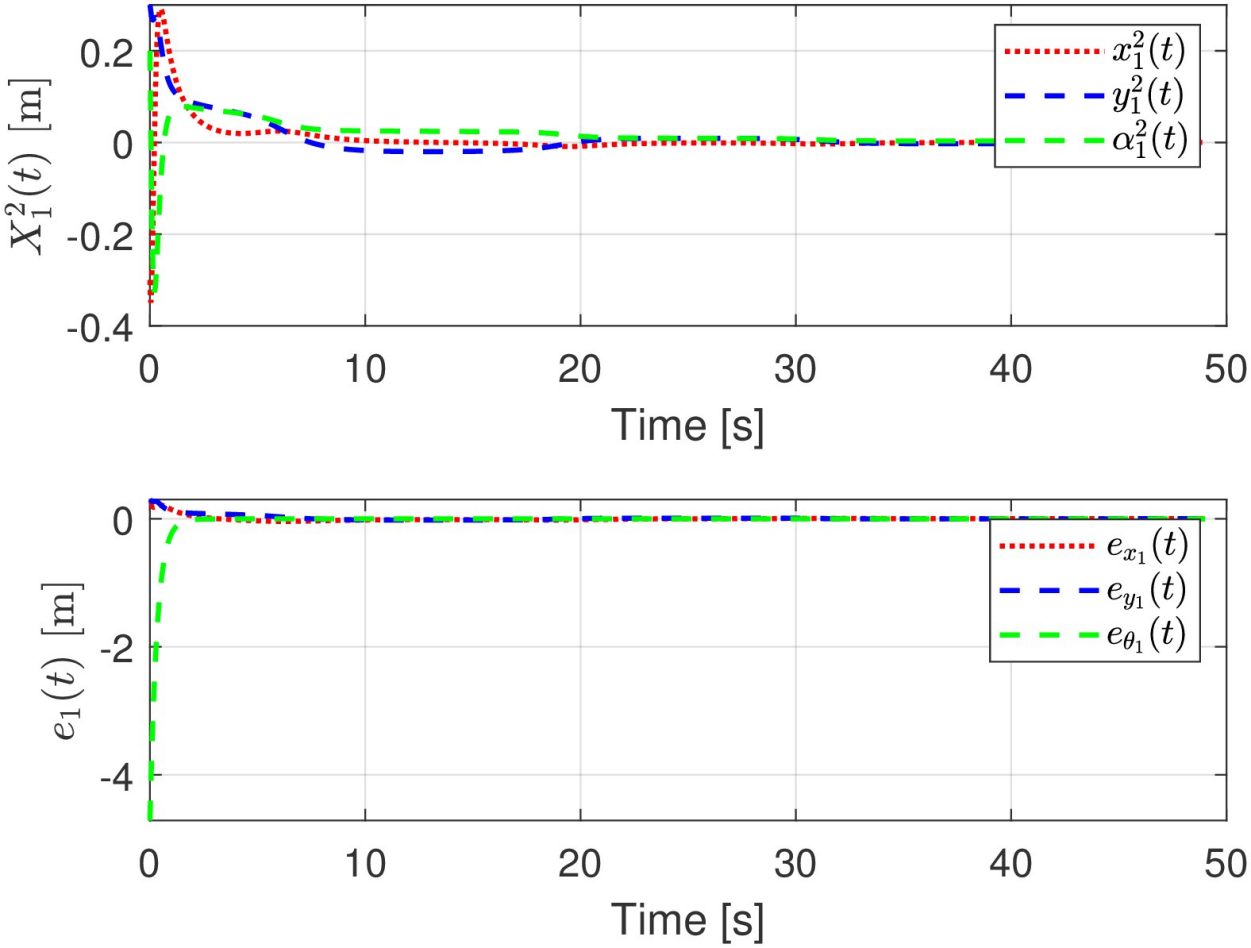
Trajectory tracking errors, moving and global frame evolution.

The evolution of the controlled tracking errors $X_{1}^{2}\left(t\right)={\left[x_{1}^{2}\left(t\right),y_{1}^{2}\left(t\right),\alpha_{1}^{2}\left(t\right)\right]}^{T}$ on the moving frame, are shown in Fig 6. In the same figure, assuming measurements on the global reference frame, the trajectory tracking errors $e_{1}\left(t\right)={\left[e_{x_{1}}\left(t\right),e_{y_{1}}\left(t\right),e_{\theta_{1}}\left(t\right)\right]}^{T}$ are depicted. Notice that these errors are not required for control implementation and are shown for complement purposes. Notice how the global and the relative error dynamics converge to zero.

**Fig 7:**
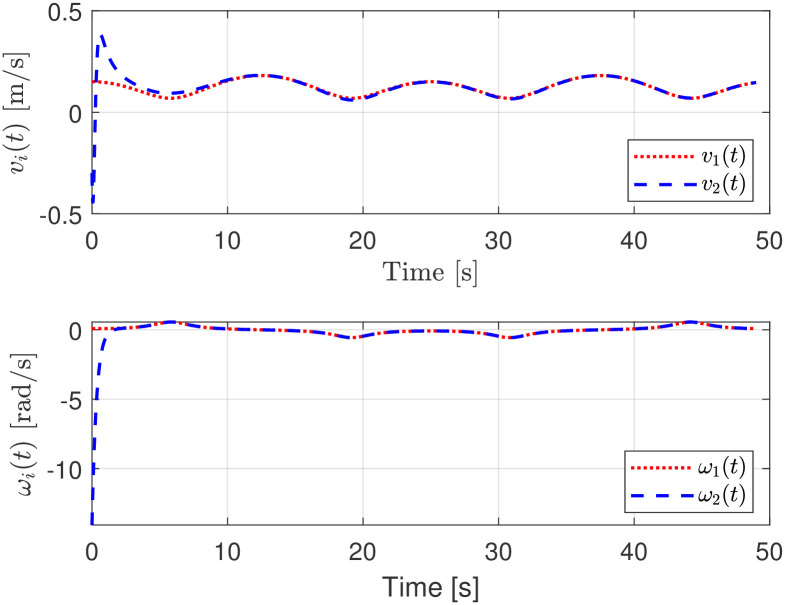
Linear and rotational velocities evolution.

The evolution of the input signals for the virtual and the actual robot is depicted in Fig 7 where its boundedness is evident.

### Numerical chain formation evaluation

For the chain formation problem, it was considered again, the bounded linear and angular velocities (33) that produce a lemniscate type trajectory for the leader robot *R*_1_. It is considered a time delay of *τ* = 3 s. between robots *R*_1_ and *R*_2_, and between robots *R*_2_ and *R*_3_. In fact, the delay time between robots does not have to be the same along the chain, but is is considered so for the sake of simplicity. The initial conditions of the formation are given in Table 2 for the global and moving frames. The gains considered for the feedback (30) with respect to *R*_2_ and *R*_3_ were set as *k*_2*i*_ = *k*_3*i*_ = 2 for *i* = 1, 2, 3.

**Table 2:** Initial conditions for the chain formation.

	Robot *R*_1_	Robot *R*_2_	Robot *R*_3_
Global Frame	*x*_1_ = 0 *y*_1_ = 0 *θ*_1_ = 0	*x*_2_ = -0.1 *y*_2_ = -0.1 $\theta_{2}=\frac{\pi }{2}$	*x*_3_ = −0.1 *y*_3_ = 0.1 $\theta_{3}=\frac{\pi }{2}$
Moving Frame *S*_2_, *S*_3_	$x_{1}^{2}=0.1$ $y_{1}^{2}=-0.1$ $\alpha_{1}^{2}=-\frac{\pi }{2}$	$x_{2}^{3}=-0.2$ $y_{2}^{3}=0$ $\alpha_{2}^{3}=0$	

**Fig 8:**
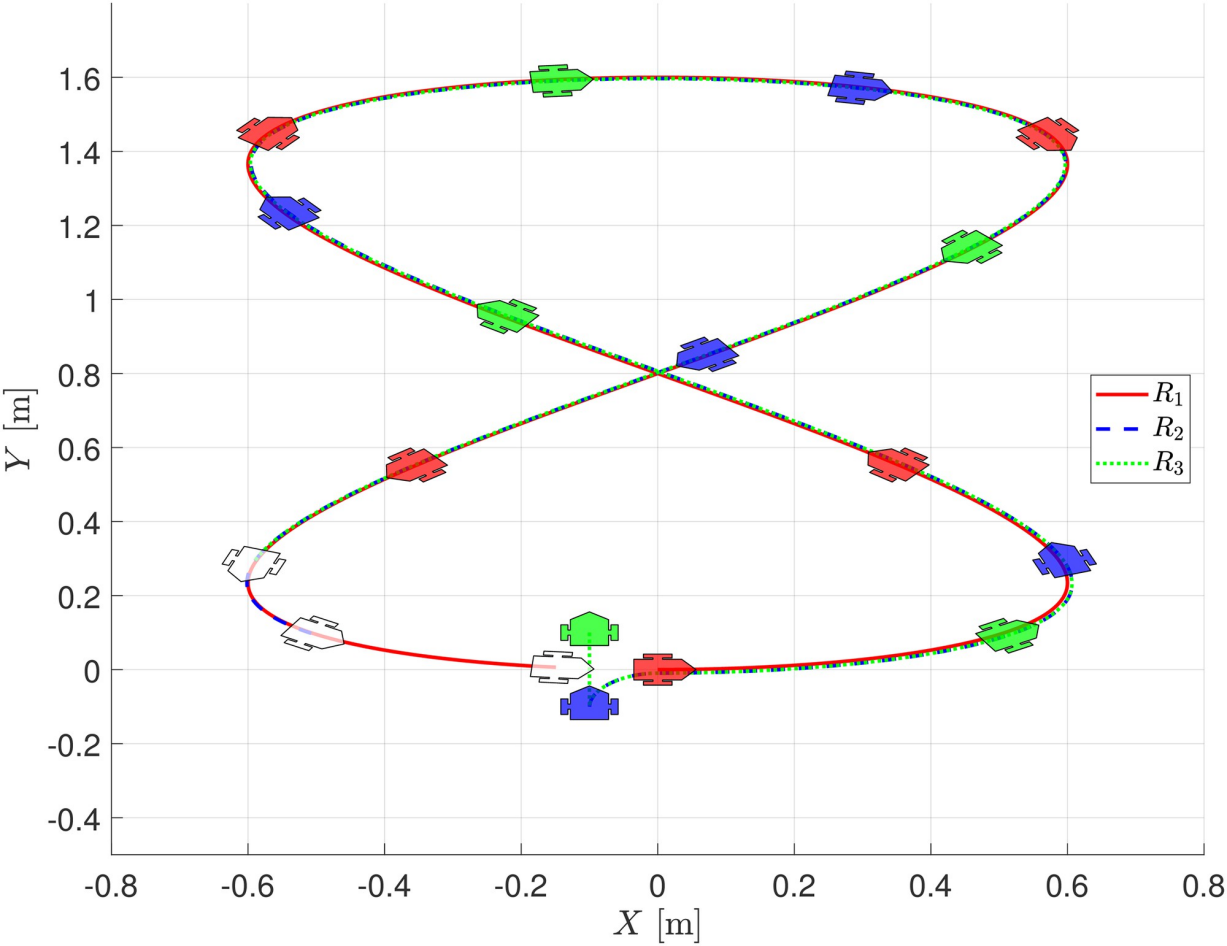
Evolution of the chain formation on the *X* − *Y* plane, numerical evaluation.

Assuming that it is possible to measure the displacements of the robots on the global reference frame *X* − *Y*, Fig 8 shows the evolution of the robots, where it is possible to see how the distance between any pair of consecutive robots changes its magnitude depending on the velocities of the robots since the formation strategy is based on a separation time between each pair of consecutive robots. Robot *R*_1_ is depicted in red, *R*_2_ in blue, and *R*_3_ in green. The final position of the robots is shown in white.

The evolution of the estimated relative distance between a pair of consecutive robots and its delayed position by means of Eq (26),

$$\begin{array}{cc} X_{1}^{1_{\tau }}\left(t\right) & ={\left[x_{1}^{1_{\tau }}\left(t\right),y_{1}^{1_{\tau }}\left(t\right),\alpha_{1}^{1_{\tau }}\left(t\right)\right]}^{T}\\ X_{2}^{2_{\tau }}\left(t\right) & ={\left[x_{2}^{2_{\tau }}\left(t\right),y_{2}^{2_{\tau }}\left(t\right),\alpha_{2}^{2_{\tau }}\left(t\right)\right]}^{T} \end{array}$$

are shown in Fig 9.

**Fig 9:**
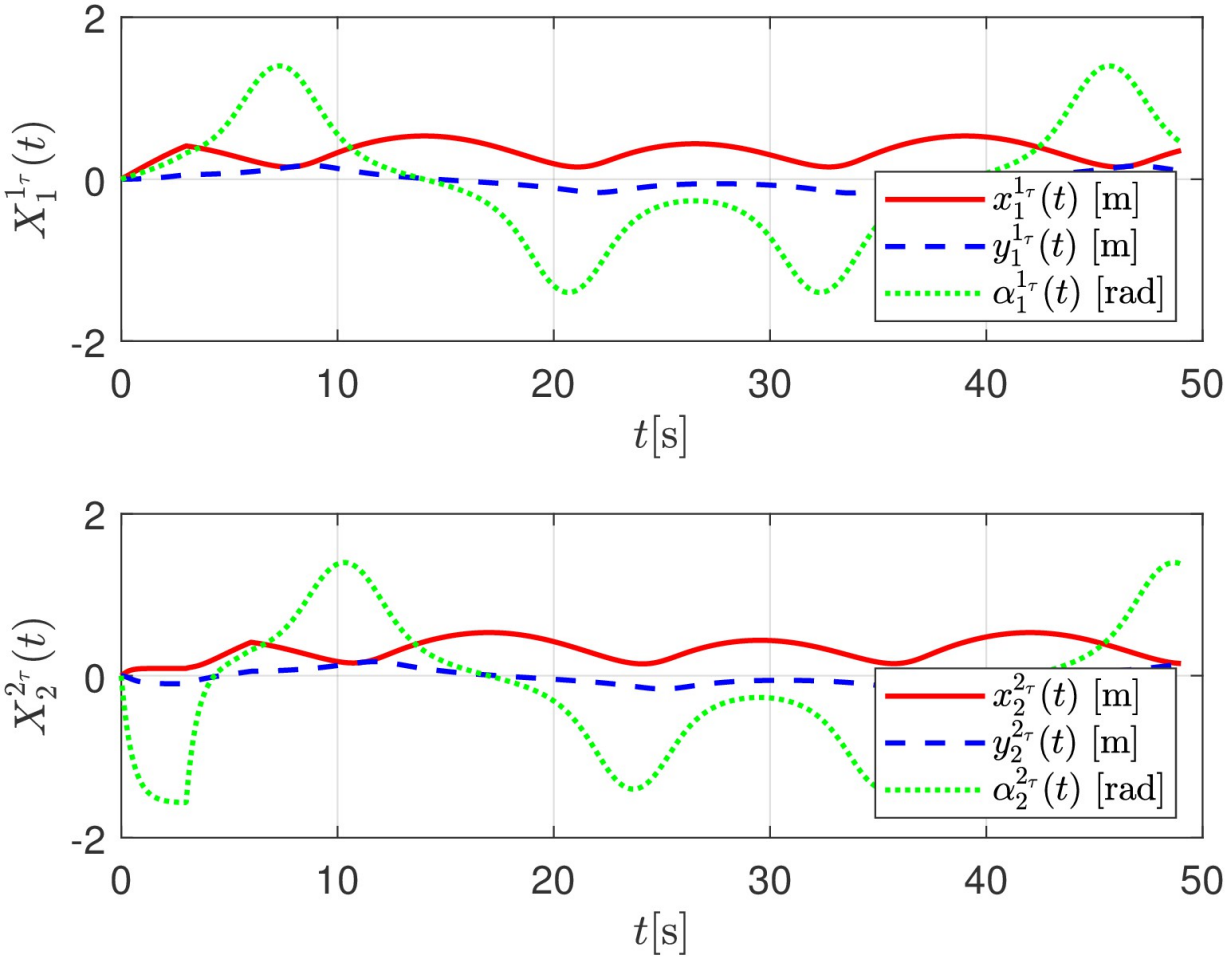
Relative distance evolution among robots, numerical evaluation.

**Fig 10:**
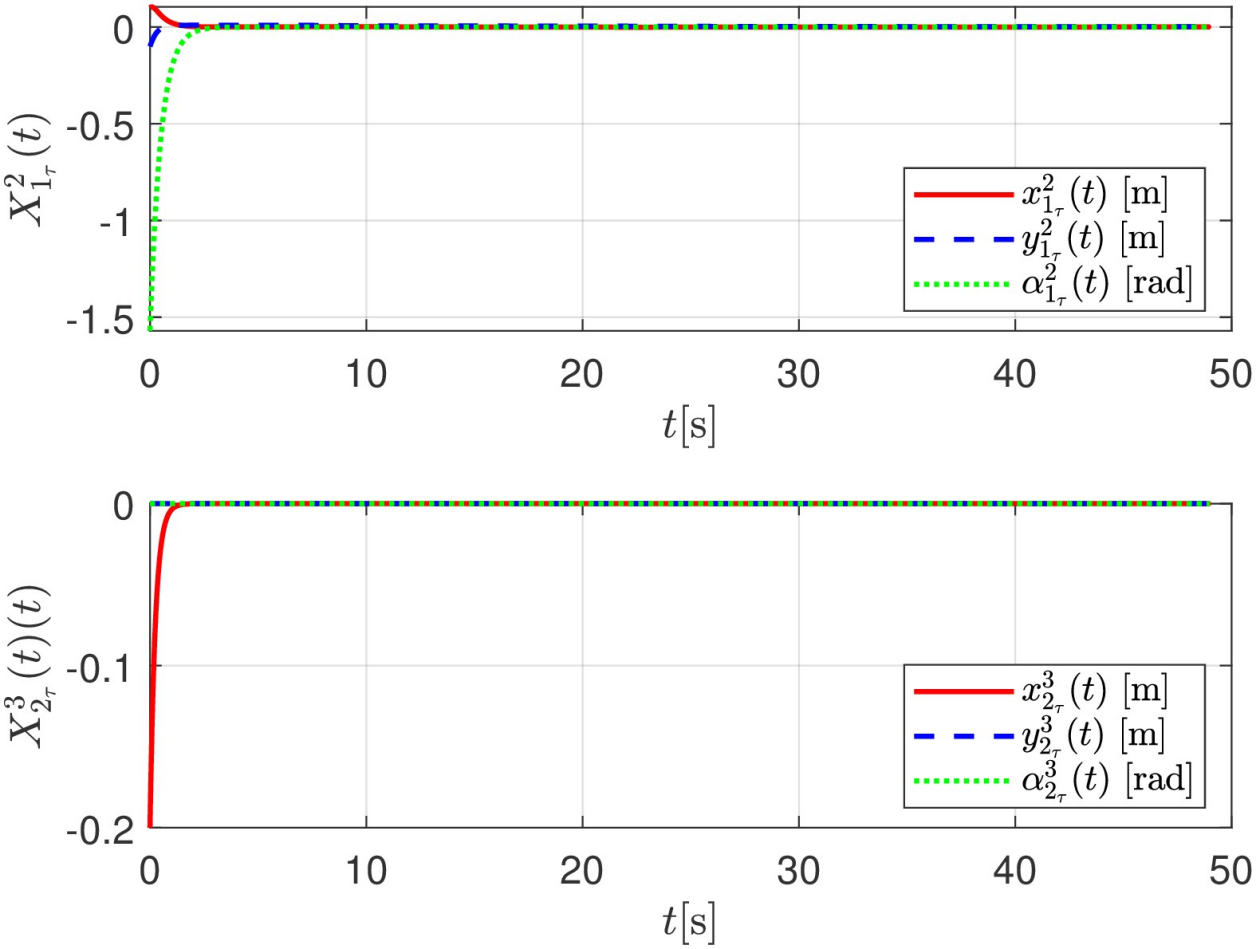
Relative trajectory tracking errors $X_{1_{r}}^{2}$ and $X_{2_{r}}^{3}$, numerical evaluation.

The convergence of the follower robot *R*_*i*+1_ to the estimated delayed trajectory of the leader robot *R*_*i*_, given as $X_{i_{\tau }}^{i+1}$, is shown in Fig 10.

**Fig 11:**
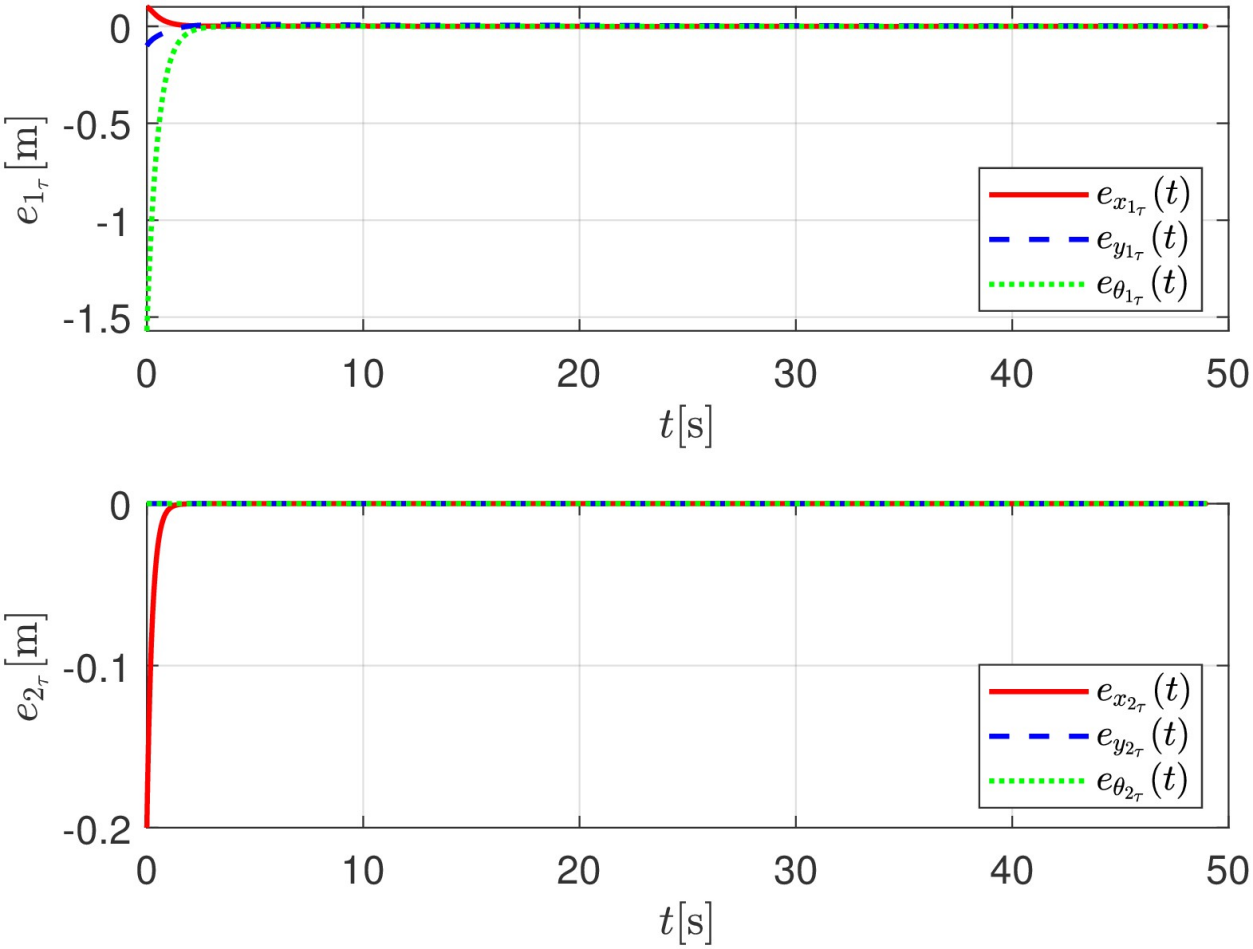
Error formation with respect to the respective delayed leader, numerical simulation.

For the sake of completeness, Fig 11 shows, on the global reference fixed frame, the convergence of the position of the follower robot *R*_*i*+1_(*t*) to the delayed trajectory of the leader robot $R_{i_{\tau }}\left(t\right)=R_{i}\left(t-\tau \right)$ by means of the error signals,

$$\begin{array}{cc} e_{1_{\tau }}\left(t\right) & ={\left[\begin{array}{ccc} e_{x_{1_{\tau }}}\left(t\right) & e_{y_{1_{\tau }}}\left(t\right) & e_{\theta_{1_{\tau }}}\left(t\right) \end{array}\right]}^{T}\\ & ={\left[\begin{array}{ccc} x_{1}\left(t-\tau \right)-x_{2}\left(t\right) & y_{1}\left(t-\tau \right)-y_{2}\left(t\right) & \theta_{1}\left(t-\tau \right)-\theta_{2}\left(t\right) \end{array}\right]}^{T} \end{array}$$

and

$$\begin{array}{cc} e_{2_{\tau }}\left(t\right) & ={\left[\begin{array}{ccc} e_{x_{2_{\tau }}}\left(t\right) & e_{y_{2_{\tau }}}\left(t\right) & e_{\theta_{2_{\tau }}}\left(t\right) \end{array}\right]}^{T}\\ & ={\left[\begin{array}{ccc} x_{2}\left(t-\tau \right)-x_{3}\left(t\right) & y_{2}\left(t-\tau \right)-y_{3}\left(t\right) & \theta_{2}\left(t-\tau \right)-\theta_{3}\left(t\right) \end{array}\right]}^{T}. \end{array}$$

**Fig 12:**
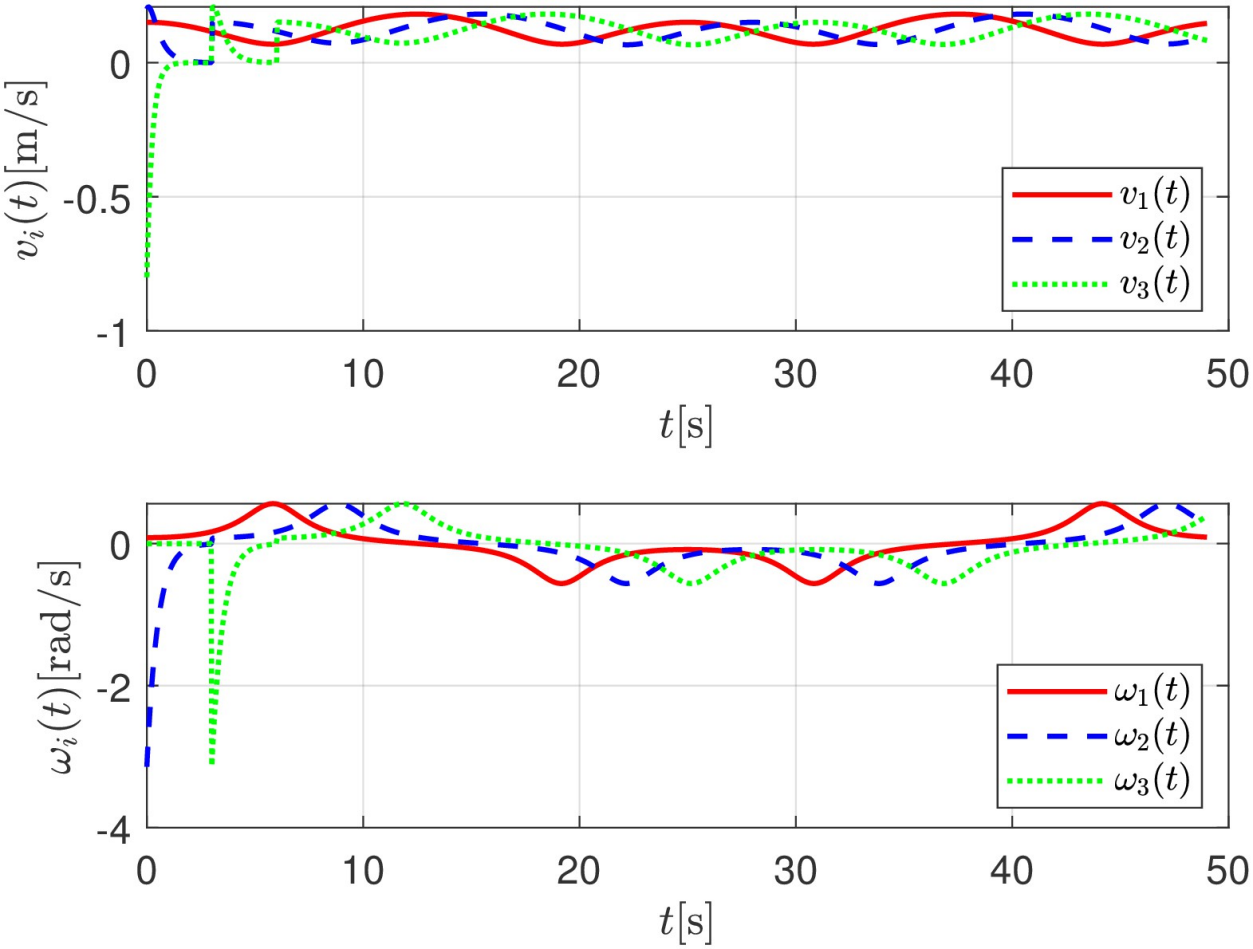
Linear *v*_*i*_(*t*) and angular *ω*_*i*_(*t*) control signals, numerical evaluation.

Finally, the set of linear *v*_*i*_(*t*) an angular velocities *ω*_*i*_(*t*) applied to the robots are depicted in Fig 12.

### Real–time chain formation evaluation

To evaluate the proposed leader–follower formation strategy, it is considered an experimental platform to carry out a real-time experiment. It is considered a set of three differentially driven mobile robots TurtleBot3 type Burger and Waffle Pi, equipped with a Raspberry Pi Model B and wireless communication.

As mentioned before, the leader robot, under bounded input velocities, provides the trajectory that the follower robots in the formation should track. The delayed trajectory of robot *R*_*i*_ that represents the desired trajectory for robot *R*_*i*+1_ is obtained by means of the virtual dynamics (26) that only requires the delayed values of the linear and angular velocities. The Optitrack vision localization system, that is used to get the relative distance and angle between a pair of consecutive robots, considers on each robot four passive markers (reflective) that are used to obtain their geometrical centroid and to compute its position and orientation on the *X* − *Y* plane. The considered indoor localization system is located on the roof of the laboratory covering a working area of 4 *m*^2^ and consists of a set of 4 Flex-13 cameras with an image resolution of 1280 × 1024, and 120 frames per second (FPS). Additionally, to the image sensors, each camera has an IR LED ring, which is reflected to the camera image sensor, obtaining in this form, the position and orientation of all the robots by using the software Motive. The pose of each robot is sent to a PC where the data is used to obtain the feedback formation strategy sent to the robots. Signals are sent by wireless communication through a VRPN (virtual reality peripheral network) and software ROS (robot operating system) that serves as a link between robots and devices.

**Remark 8** *It should be pointed out that although on the real time experiments it has been considered an indoor global positioning system (Optitrack), this is not necessary to carry out the evaluation. It could be possible to consider a simple on board monocular camera mounted on the follower robots to get the relative distance and angular error in the way that it is proposed is* [28]. *Other options are the used of Ultra Wide Band (UWB) based radio transceivers, such as in* [29], *or* [30], *where they also integrated UWB with an IMU and camera onboard*.

The initial condition for the experiments are shown in Table 3 while the gains on the feedback law (30) for each pair of consecutive robots were set to *k*_11_ = 0.2, *k*_12_ = 15, *k*_13_ = 0.2 and *k*_21_ = *k*_31_ = 0.2, *k*_22_ = *k*_32_ = 25 and *k*_23_ = *k*_33_ = 3.5.

**Table 3:** Experimental initial conditions.

	Robot *R*_1_	Robot *R*_2_	Robot *R*_3_
Global Frame	*x*_1_ = 0.017 *y*_1_ = -0.707 *θ*_1_ = -0.187	*x*_2_ = −0.315 *y*_2_ = −0.628 *θ*_2_ = −0.018	*x*_3_ = −0.299 *y*_3_ = −0.813 *θ*_3_ = 0.107
Moving Frame *S*_2_, *S*_3_	$x_{1}^{2}=0.333$ $y_{1}^{2}=-0.072$ $\alpha_{1}^{2}=-0.166$	$x_{2}^{3}=0.004$ $y_{2}^{3}=0.185$ $\alpha_{2}^{3}=-0.089$	

**Fig 13:**
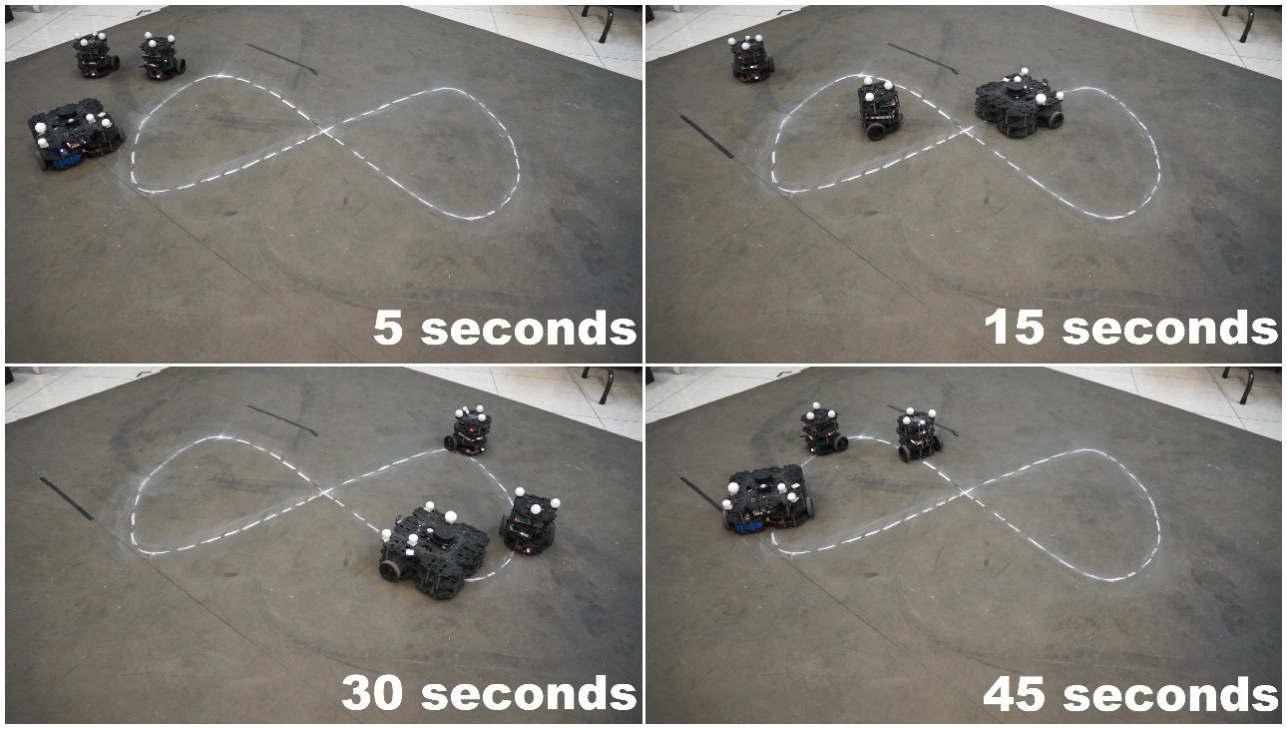
Real-time displacement of the vehicles over a Lemniscate type path.

**Fig 14:**
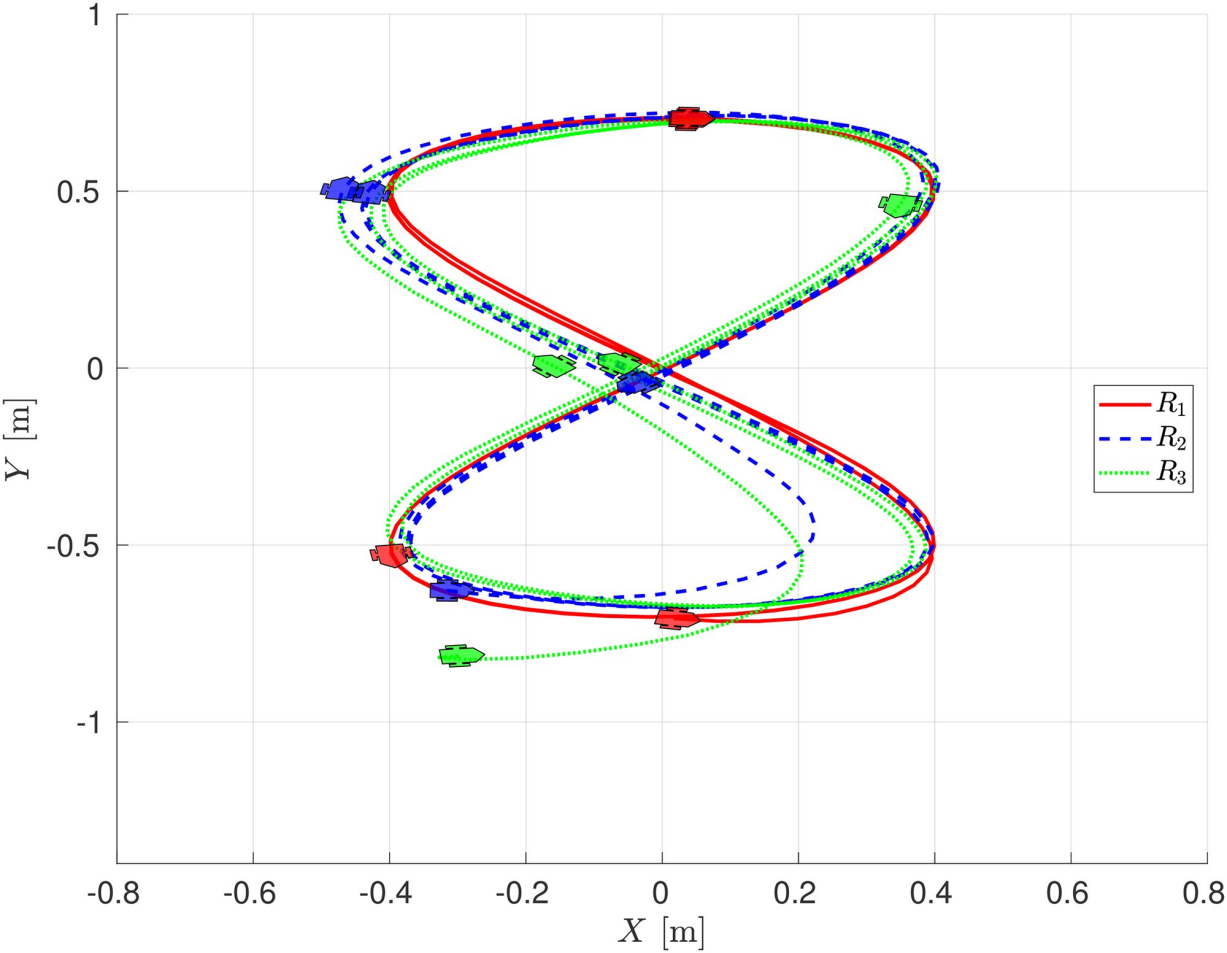
Evolution of the chain formation on the *X* − *Y* plane, experimental evaluation.

A snapshot of the experiment is shown in Fig 13 where four moments of the experiments are depicted, while Fig 14 shows the evolution of the robot on the *X* − *Y* plane, showing an adequate convergence of each follower robot to the trajectory described by its leader robot, this is, robot *R*_2_ follows the delayed trajectory of robot *R*_1_, and robot *R*_3_ follows the corresponding one of robot *R*_2_.

**Fig 15:**
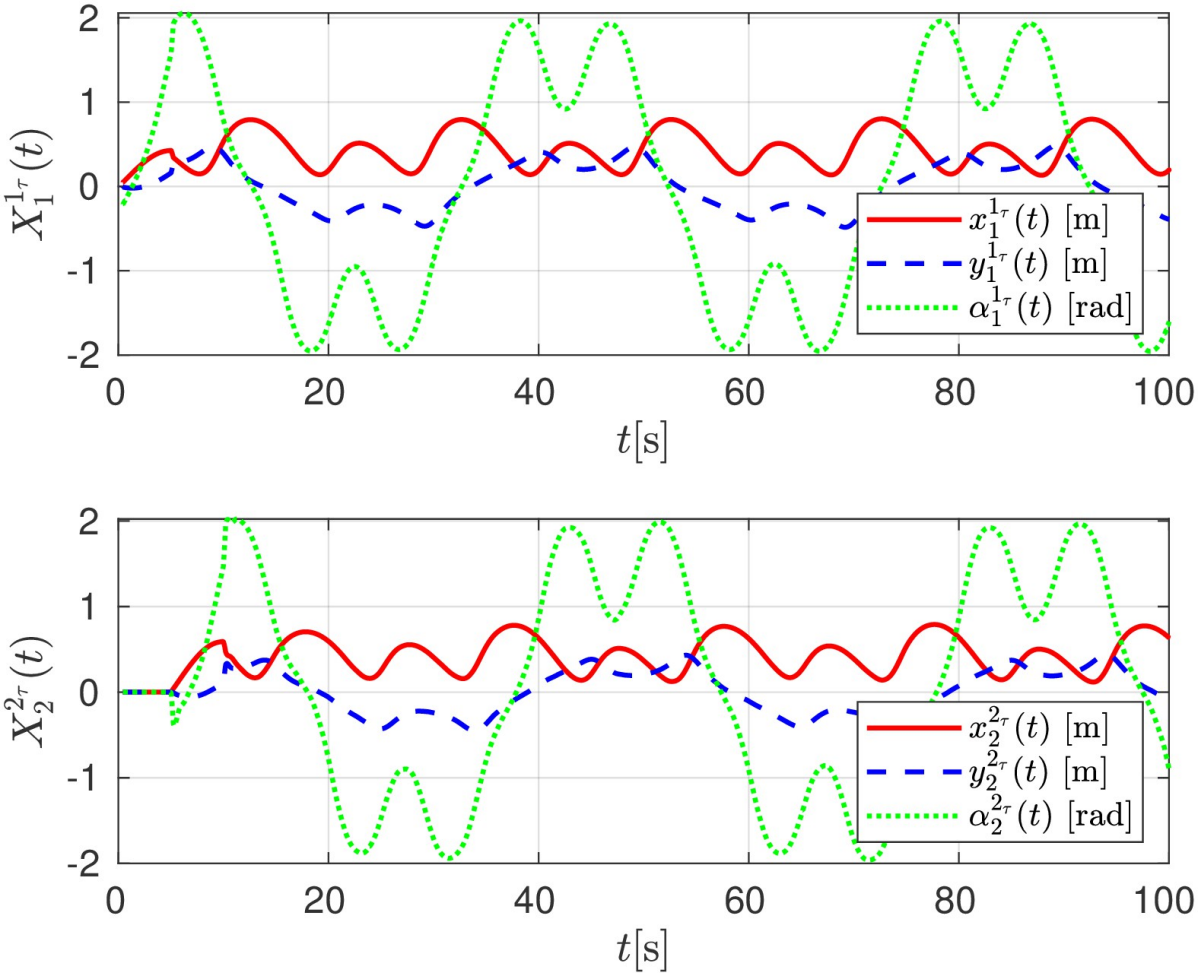
Relative distance evolution among robots, experimental evaluation.

**Fig 16:**
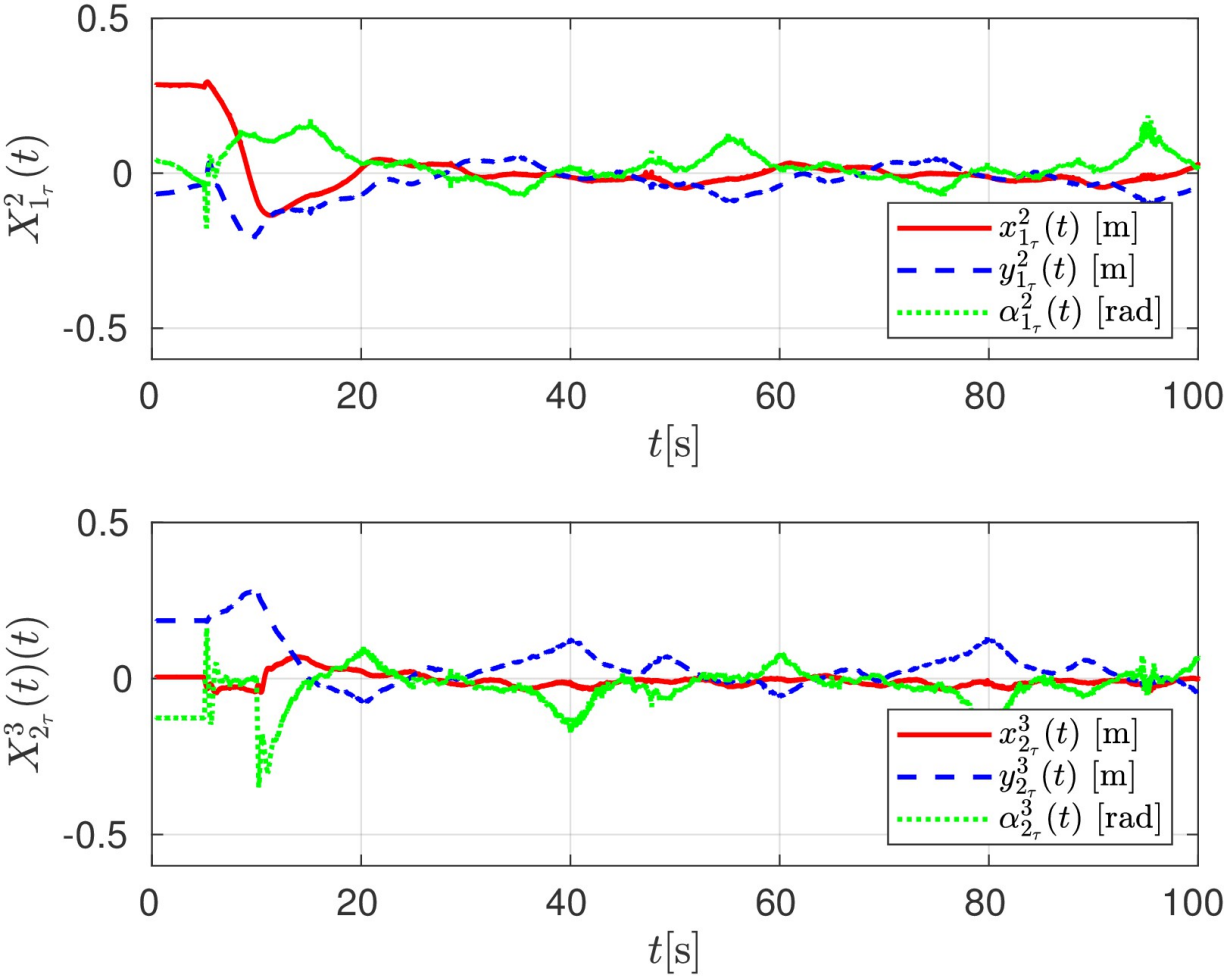
Relative trajectory tracking errors $X_{1_{r}}^{2}$ and $X_{2_{r}}^{3}$, experimental evaluation.

The relative distance between any robot and its delayed position $X_{1}^{1_{\tau }}\left(t\right)$, $X_{2}^{2_{\tau }}\left(t\right)$ are shown in Fig 15. The convergence of the follower robot *R*_*i*+1_ to the estimated delayed trajectory of the leader robot *R*_*i*_, given as $X_{i_{\tau }}^{i+1}$, is shown in Fig 16.

**Fig 17:**
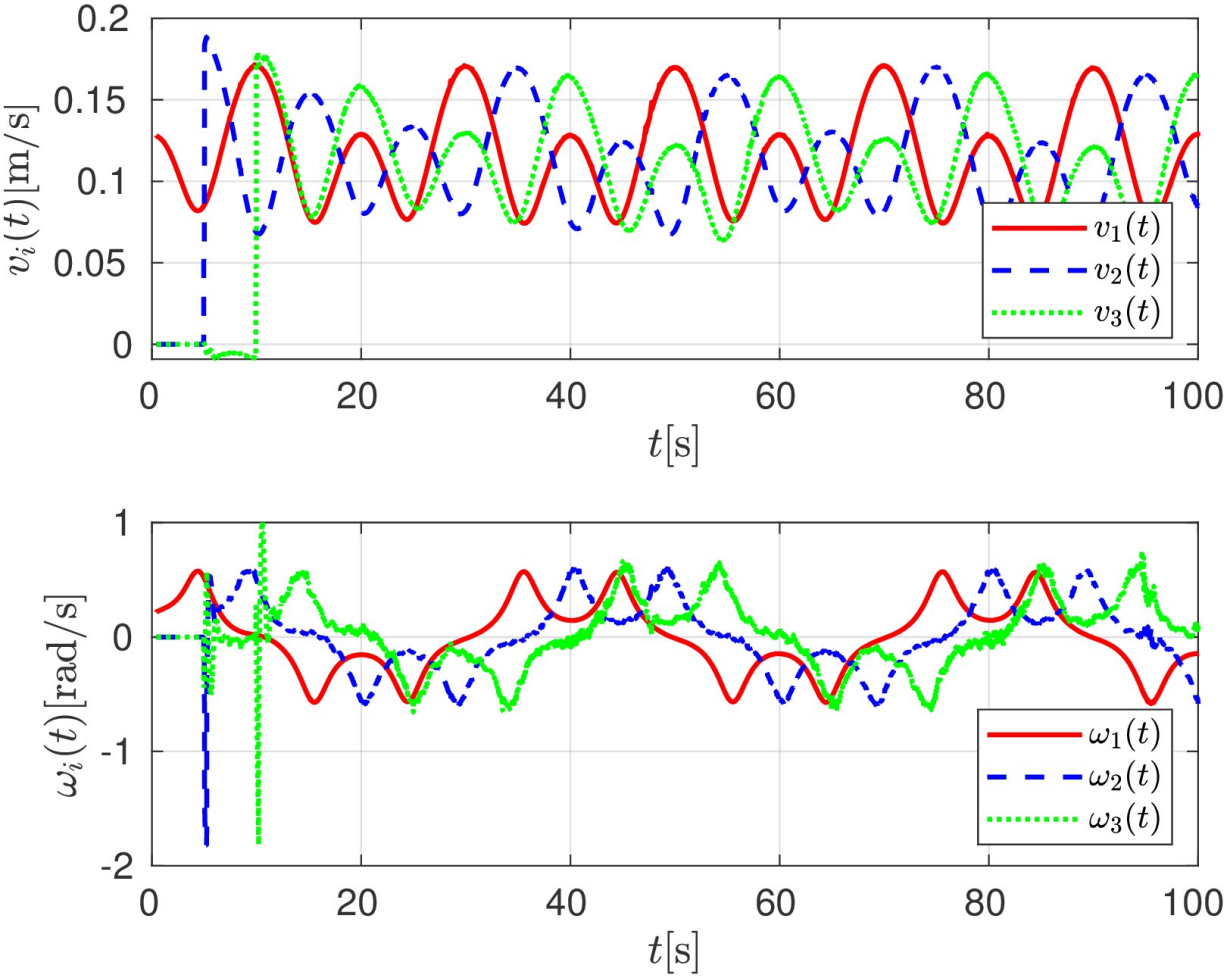
Linear *v*_*i*_(*t*) and angular *ω*_*i*_(*t*) control signals, experimental evaluation.

The linear *v*_*i*_(*t*) and angular velocities *ω*_*i*_(*t*) applied to the robots are depicted in Fig 17.

**Remark 9** *It should be pointed out that the proposed formation strategy does not consider a collision avoidance strategy. This fact could represent and additional problem related with the convergence of the robots to their position in the formation. Since the desired trajectory of robot R*_*i*+1_ *is given by the delayed trajectory of robot R*_*i*_ *obtained by* (26), *it is clear that robot R*_*i*+1_ *starts moving τ units of time after the movement of robot R*_*i*_. *This fact, together with appropriate initial conditions avoid the collision of the robots in the formation. Of course, this problem has to be analyzed as a future work*.

## Conclusions

This work has presented a solution for a chain formation problem for a set of *n* differential drive mobile robots. The solution strategy is developed considering local moving frames located on the middle point of the axis wheel of the robots. The considered solution allows avoidance of the use of a global reference frame that restricts, in general, the working space of the formation. It is proposed that the *R*_*i*+1_ robot in the formation tracks the delayed trajectory, *τ* units of time, of the *R*_*i*_ robot. This delayed desired trajectory is generated by the knowledge of the input velocities of the *R*_*i*_ and *R*_*i*+1_ robot. The strategy is formally proved by means of a Lyapunov approach. The evaluation of the control proposal is carried out by means of numerical simulations and real time experiments, showing an adequate convergence of robot *R*_*i*+1_ to its desired trajectory achieving the platoon formation.
